# Genome-wide association study of prolactin levels in blood plasma and cerebrospinal fluid

**DOI:** 10.1186/s12864-016-2785-0

**Published:** 2016-06-29

**Authors:** Lyndsay A. Staley, Mark T. W. Ebbert, Sheradyn Parker, Matthew Bailey, for the Alzheimer’s Disease Neuroimaging Initiative, Perry G. Ridge, Alison M. Goate, John S. K. Kauwe

**Affiliations:** Department of Biology, Brigham Young University, Provo, UT 84602 USA; Biology and Biomedical Sciences, Washington University, St. Louis, MO 63110 USA; Department of Neuroscience Icahn School of Medicine, New York, NY 10029 USA

**Keywords:** Association, Genetics, CSF, Plasma, Prolactin

## Abstract

**Background:**

Prolactin is a polypeptide hormone secreted by the anterior pituitary gland that plays an essential role in lactation, tissue growth, and suppressing apoptosis to increase cell survival. Prolactin serves as a key player in many life-critical processes, including immune system and reproduction. Prolactin is also found in multiple fluids throughout the body, including plasma and cerebrospinal fluid (CSF).

**Methods:**

In this study, we measured prolactin levels in both plasma and CSF, and performed a genome-wide association study. We then performed meta-analyses using METAL with a significance threshold of *p* < 5 × 10^−8^ and removed SNPs where the direction of the effect was different between the two datasets.

**Results:**

We identified 12 SNPs associated with increased prolactin levels in both biological fluids.

**Conclusions:**

Our efforts will help researchers understand how prolactin is regulated in both CSF and plasma, which could be beneficial in research for the immune system and reproduction.

**Electronic supplementary material:**

The online version of this article (doi:10.1186/s12864-016-2785-0) contains supplementary material, which is available to authorized users.

## Background

Prolactin, a hormone mostly secreted from the lactotroph cells within the anterior pituitary gland [1] and expressed by the *PRL* gene, plays an important role in milk lactation for pregnant women [1], helps regulate the menstrual cycle, and also affects reproduction, metabolism, homeostasis, tissue growth, osmoregulation, immunoregulation, and behavior [2, 3]. Prolactin levels are regulated in a short-loop feedback mechanism by prolactin inhibitory factors (PIF), dopamine being an important example [4]. This feedback system changes during pregnancy, and prolactinomas, hypothyroidism, medications, stress, exercise, herbs, and certain foods can also affect prolactin levels [5, 6]. Prolactin has also been shown to suppress apoptosis, and increase survival and function of cells, including T-lymphocytes [7].

Cerebrospinal fluid (CSF) and plasma separated by the blood–brain barrier and levels of expression in these biological fluids are often independent, suggesting the genes are regulated independently across tissues on either side of the blood–brain barrier [8]. Currently, little is known about genetic markers that affect prolactin expression in plasma or CSF. In this study we conducted a genome-wide association study of prolactin levels in the CSF and in the plasma of individuals from two datasets, looking for SNPs that are associated with prolactin levels in both CSF and plasma. Further research of the variants we identified will help researchers further understand how prolactin is regulated across multiple tissues in the human body and how it affects human health.

## Methods

### Subjects and data description

CSF and plasma samples were collected from the Knight-Alzheimer’s Disease Research Center at Washington University School of Medicine (Knight ADRC) and from the Alzheimer’s Disease Neuroimaging Initiative (ADNI). In this study, we used 297 CSF and 347 plasma samples from ADNI, and 246 CSF and 240 plasma samples from Knight ADRC. The majority of the samples were controls, although 7 % of Knight ADRC samples were Alzheimer’s disease cases, and 15 % of ADNI samples were AD cases. Levels for 190 biomarkers were measured for each sample using the Human DiscoveryMAP Panel v1.0 and a Luminex 100 platform [9] and the samples were genotyped using the Illumina 610 or the Omniexpress chip. A description of the collection methods and the Knight ADRC samples has been previously published [10, 11] and the ADNI samples were collected as part of the ADNI biomarker study [12], and were obtained from the ADNI database (adni.loni.usc.edu). All samples were of European descent, and varied in age from 58 to 91 years, with an average age of 76 years, for the ADNI samples, and varied in age from 49 to 91 years, with an average age of 73 years, for the Knight ADRC samples. All individuals whose data were included in this study were explicitly consented, following appropriate Institutional Review Board policies.

### SNP imputation

SNPs were imputed as previously described [13]. Beagle was used to impute SNPs from the data from the 1000 Genomes Project (June 2012 release). Imputed SNPs with the following criteria were removed: (1) an r^2^ of 0.3 or lower, (2) a minor allele frequency (MAF) lower than 0.05 (3) out of Hardy-Weinberg equilibrium (*p* < 1 × 10 − 6), (4) a call rate lower than 95 %, or (5) a Gprobs score lower than 0.90. Exactly 5,815,690 SNPs passed the QC process.

### Data cleaning and analysis

We conducted analyses using PLINK [14], a whole genome association analysis toolset. We excluded SNPs that exceeded thresholds for Hardy-Weinberg Equilibrium [15, 16] (--hwe 0.00001), missing genotype rate (--geno 0.05), and minor allele frequency (--maf 0.01) on the Knight ADRC and ADNI datasets. Then, we excluded individuals with a missing genotype rate greater than 2 % (--mind 0.02).

With the cleaned data, we conducted a linear regression for all remaining SNPs, within each data set, to test for an association with prolactin levels, adjusting for age, gender, and the first two principle components generated using EigenSoft [17, 18]. We then performed a meta-analysis across ADNI and Knight ADRC for CSF and another meta-analysis across ADNI and Knight ADRC for plasma, each accounting for sample size, *p*-values, and direction of effect using the default METAL [19] settings.

We retained all SNPs that had a meta-analysis p-value less than 5 × 10^−8^ and that had the same direction of effect in both the Knight ADRC and ADNI datasets, in both resulting meta-analysis files. We then looked for SNPs that were replicated in both the significant CSF and plasma meta-analysis resulting files. We searched for these SNPs in the NHGRI catalog of published genome-wide association studies [20]. (downloaded October 12th, 2015) for known disease associations. We then used RegulomeDB [21] and functional annotations from wAnnovar [22, 23] to identify SNPs that are biologically likely to modify gene function or expression. RegulomeDB scores range from “1a” to “6”. Lower scores indicate stronger evidence that the SNP affects gene regulation based on both empirical data, such as ChIP-seq, and whether the SNP is within a known transcription factor binding motif. We generated regional association plots using SNAP [24] for regions of interest and explored whether any genes of interest are part of the same pathway or regulatory network using PathwayCommons [25]. For SNPs where linkage disequilibrium data is unknown in SNAP, we modified the SNAP source code to plot all SNPs in the region regardless of linkage disequilibrium status and omit r^2^ values. By default, SNAP only plots SNPs with a known r^2^ greater than 0. We also generated q-q plots in R to check for evidence of inflation of *p*-values.

## Results

**Fig. 1:**
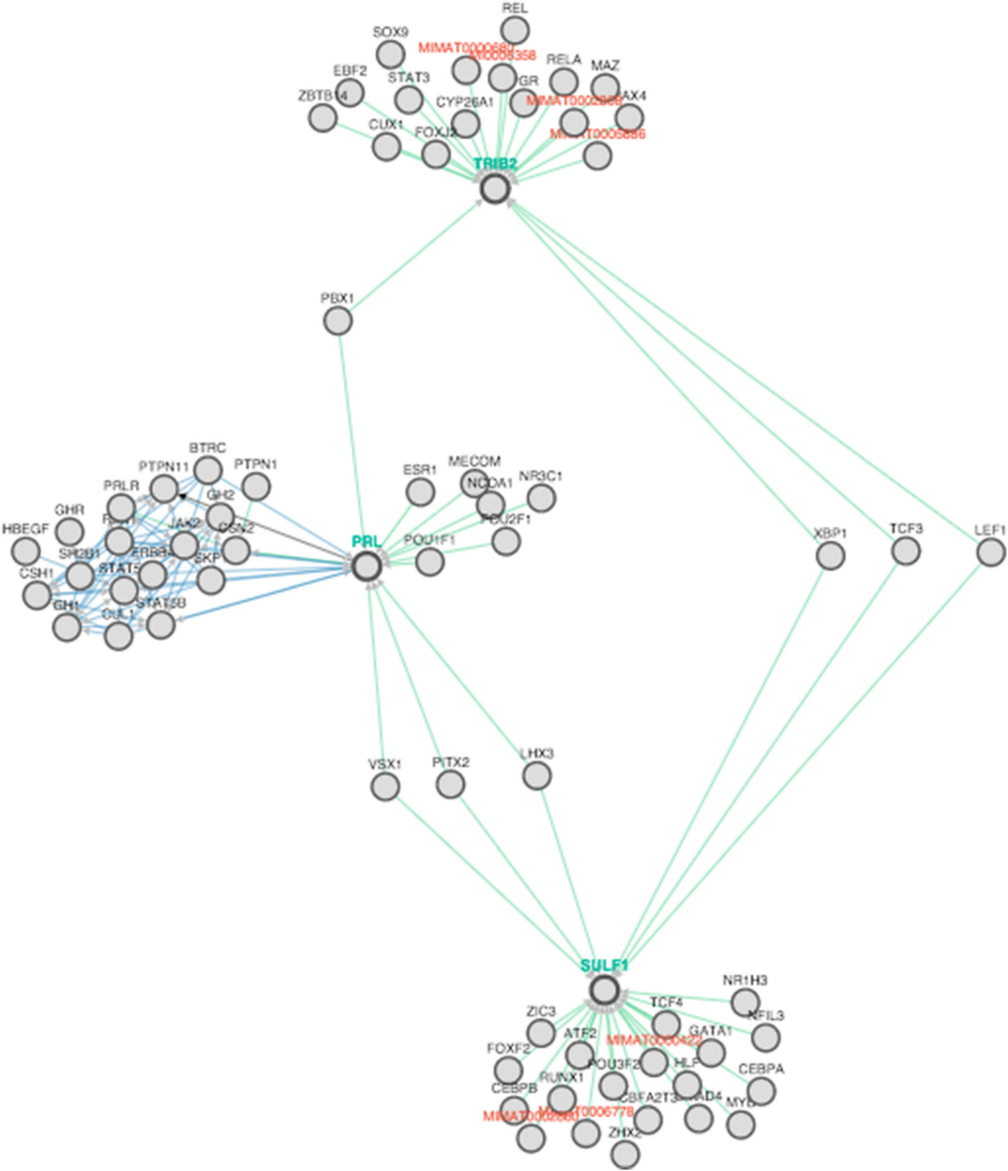
PathwayCommons output showing the gene that codes for prolactin along with the major players SULF1 and TRIB2. Our significant SNPs, rs12548348 and rs13408093, fall in SULF1 and TRIB2, respectively. This means that although none of the SNPs fall directly in or near PRL, they could still be affecting the prolactin pathway because they are regulated by some of the same transcription factors

We identified 37 SNPs associated with prolactin levels in plasma and 666 SNPs associated with prolactin levels in CSF (Additional files 1 and 2), none of which are located in or around the *PRL* gene. Significant SNPs were spread across 21 chromosomes for the CSF results and across 10 different chromosomes for the plasma results. There are several hits on chromosome 6, but all are more than 5 million base pairs away from where the *PRL* gene is located. There were 12 SNPs in common between the plasma and CSF results (Table 1), 6 of which were on chromosome 6, approximately 6 million base pairs away from the *PRL* gene. RegulomeDB scores for the 12 SNPs ranged from 4 to 6 and MAFs ranged from 0.06 to 0.14. None of the 12 SNPs were found in the NHGRI catalog of published genome-wide association studies. The q-q plots demonstrated no evidence of inflation (genomic inflation factor = 1.0; Additional files 3 and 4). According to PathwayCommons, *PRL*, *SULF1*, and *TRIB2* are all regulated by some of the same transcription factors (Fig. 1) including *PBX1*, *XBP1*, *TCF3*, *LEF1*, *VSX1*, *PITX2*, and *LHX3*. There were no other known relationships among the genes identified in this study.

**Table 1:** Significant SNPs were scattered across chromosomes 2, 6, 7, 8 and 17, with the majority of the SNPs being on chromosome 6. These 12 SNPs were all significant in both the blood plasma and CSF. Information on the SNPs includes chromosome, reference and alternate allele, minor allele frequency, predicted function, the gene the SNP is found in or near, RegulomeDB score, and the meta-analysis *p*-values for plasma and CSF

SNP	Chr	Base Pair Position	Major Allele	Minor Allele	MAF	Predicted Function	Gene	RegulomeDB score	Meta-analysis *p*-value	
									Plasma	CSF
rs12548348	8	70430077	A	G	0.1222	Intronic	*SULF1*	No Data	6.288e-11	9.841e-26
rs13408093	2	62251682	A	T	0.0699	Intronic	*TRIB2*	5	6.881e-10	2.119e-25
rs1150703	6	28184260	G	T	0.0919	ncRNA_exonic	*TOB2P1*	5	3.276e-09	7.011e-26
rs988083	6	28177588	C	T	0.1220	Intergenic	*ZNF192P1*,*TOB2P1*	5	3.276e-09	7.011e-26
rs988084	6	28177492	C	T	0.1218	Intergenic	*ZNF192P1*,*TOB2P1*	6	3.276e-09	7.011e-26
rs73726888	7	15021811	T	C	0.0893	UTR3	*GIMAP7*	6	4.209e-09	6.438e-24
rs8073041	17	47498253	T	A	0.0731	Intergenic	*PHB*,*LOC101927207*	6	7.87e-09	2.169e-10
rs1150701	6	28183886	A	C	0.1410	ncRNA_exonic	*TOB2P1*	6	1.184e-08	1.443e-26
rs1150702	6	28184097	A	T	0.1410	ncRNA_exonic	*TOB2P1*	5	1.184e-08	1.443e-26
rs1233712	6	28193131	G	A	0.1406	UTR5	*ZSCAN9*	4	1.184e-08	1.443e-26
rs79268972	17	47531241	T	G	0.0749	Intergenic	*PHB*,*LOC101927207*	5	2.547e-08	8.169e-12
rs77482998	7	47532356	T	C	0.0665	Intronic	*TNS3*	5	4.608e-08	7.983e-12

## Discussion

**Fig. 2:**
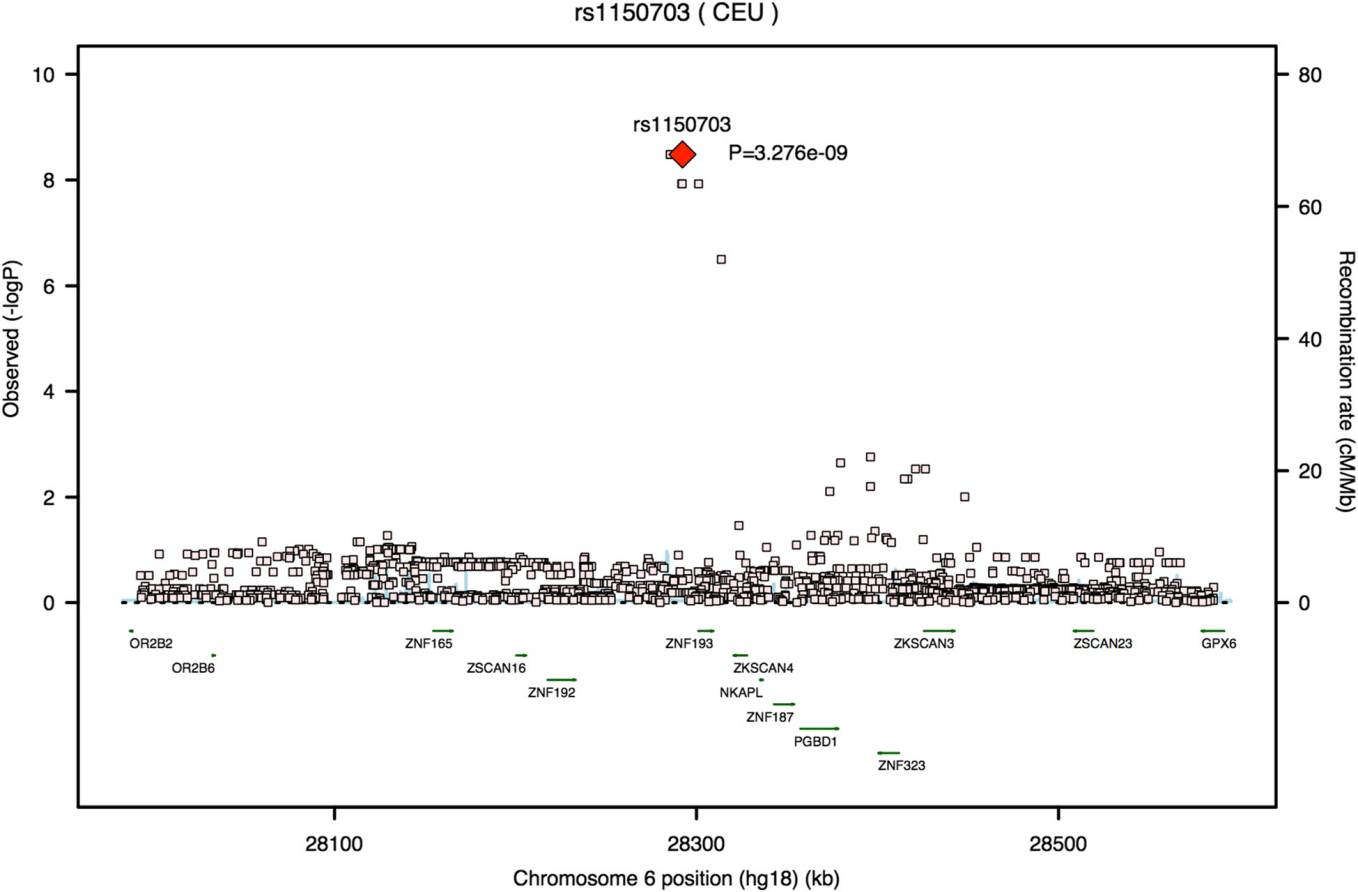
Regional association plot generated using SNAP showing rs1150703 has the strongest association with prolactin plasma levels of the SNPs found in this region of chromosome 6. We identified several SNPs associated with prolactin levels in the plasma and plotted association *p*-values in the region. We omitted r^2^ values in this plot because SNAP does not have linkage disequilibrium data for this SNP

**Fig. 3:**
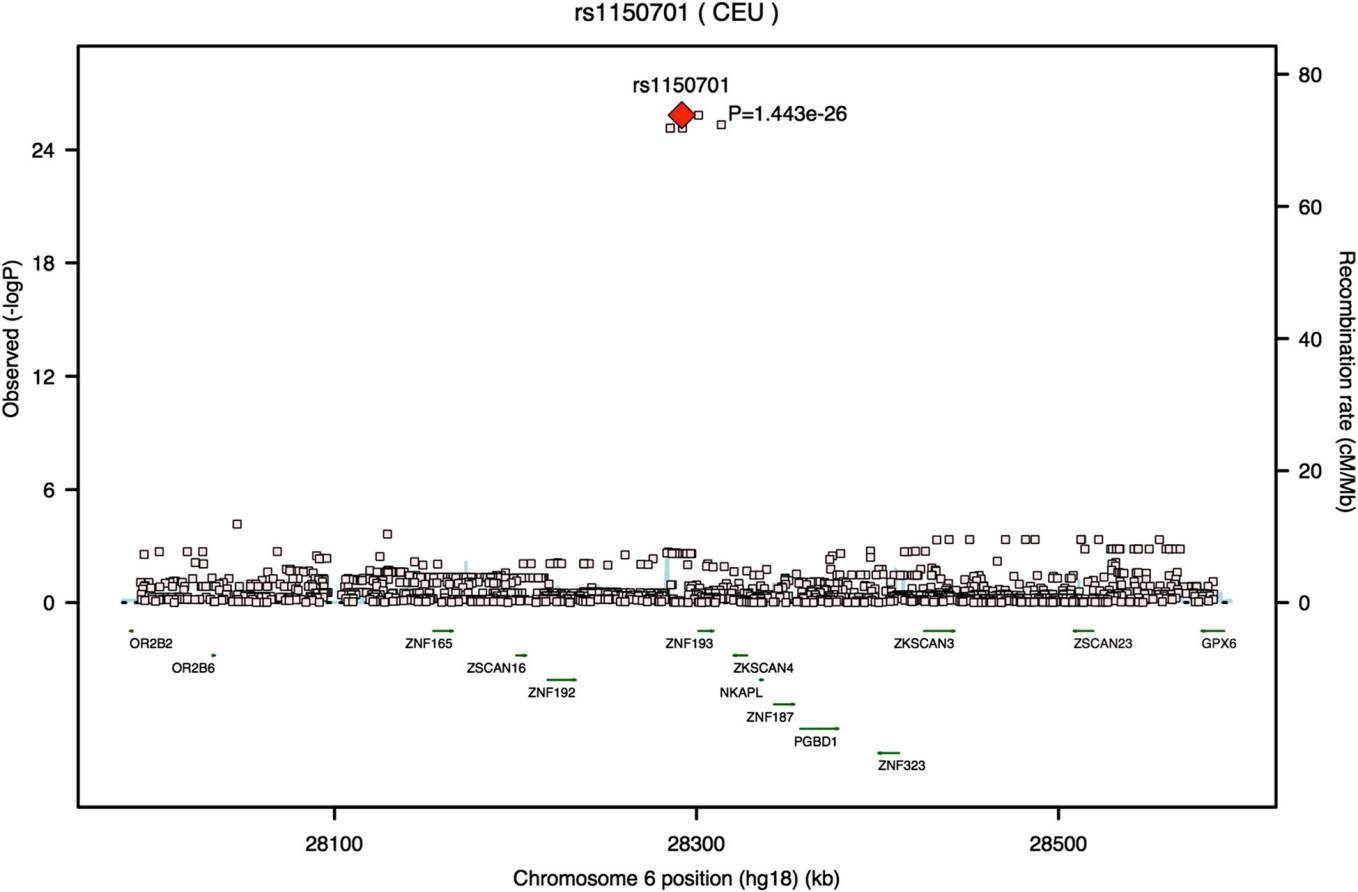
Regional association plot generated using SNAP showing rs1150701 has the strongest association with prolactin CSF levels of the SNPs found in this region of chromosome 6. We identified several SNPs associated with prolactin levels in the CSF and plotted association *p*-values in the region. We omitted r^2^ values in this plot because SNAP does not have linkage disequilibrium data for this SNP

Twelve SNPs were significantly associated with prolactin levels in both plasma and CSF, 6 are located on chromosome 6 and the remaining 6 SNPs are scattered across chromosomes 2, 7, 8, and 17. The 6 SNPs on chromosome 6 cluster in and around *ZSCAN9*, *TOB2P1*, and *ZNF192P1*, according to Annovar, though visualizing the SNPs’ locations in the NCBI viewer shows that 3 of the 6 SNPs fall within a *ZSCAN9* intron for one specific transcript (XM_011514877.1) as well as within *TOB2P1*—a pseudogene that falls within the same intronic region of *ZSCAN9*. SNP rs1233712 is in the 5′UTR region of *ZSCAN9*. SNPs rs988083 and rs988084 are between *ZNF192P1* and *TOB2P1*, according to Annovar. *ZNF192P1* is also a pseudogene that is proximal to *ZSCAN8*. In short, all 6 SNPs on chromosome 6 are located in or around *ZSCAN8* and *ZSCAN9*, both of which are protein-coding genes, while 3 of the 6 fall directly within a pseudogene (*TOB2P1*). Of the significant SNPs on chromosome 6, rs1150703 was most significantly correlated with prolactin levels in plasma (Fig. 2) while rs1150701 was most significantly correlated with prolactin levels in CSF (Fig. 3).

The remaining 6 SNPs are located on chromosomes 2, 7, 8, and 17, where 2 of the SNPs are intergenic, 3 are intronic, and one is located in a 3′UTR region (Table 1). SNP rs12548348 is an intronic SNP within the *SULF1* gene on chromosome 8 and was most significantly associated with prolactin levels in plasma out of the 12 found in common between the two fluids. It was also one of most significantly associated with prolactin levels in CSF. SNPs rs13408093 and rs77482998 are intronic SNPs within the *TRIB2* (chromosome 2) and *TNS3* (chromosome 7) genes, respectively. SNPs rs8073041 and rs79268972 are intergenic SNPs that are both located on chromosome 17 between the gene *PHB* and a non-coding RNA *LOC101927207*. The next closest protein-coding gene is *NGFR*. SNP rs73726888 is located in the 3′UTR region of *GIMAP7* on chromosome 7. While rs77482998 (*TNS3*) and rs73726888 (*GIMAP7*) are both located on chromosome 7, they are distant from each other on opposite arms of the chromosome, suggesting their associations with prolactin levels are independent of each other.

While there is no direct evidence that any of these markers directly impact prolactin expression, it appears that *PRL*, *SULF1*, and *TRIB2* in that they are all regulated by common transcription factors, including *PBX1*, *XBP1*, *TCF3*, *LEF1*, *VSX1*, *PITX2*, and *LHX3*. It is possible that these genes and variants are involved in *PRL* regulation through more complex biological relationships. This may be significant because genes regulated by the same transcription factor are often active in the same tissues at the same time [26, 27].

## Conclusions

In summary, we have identified significant and replicable association between several genetic variants in both plasma and CSF levels of prolactin. These results provide a foundation for a better understanding of prolactin regulation, and in turn the host of phenotypes in which prolactin plays a role, including lactation, immunoregulation, apoptosis and T-lymphocyte function [1–3, 7]. Future work on these associated markers will provide meaningful insights into these phenotypes.

## Abbreviations

PIF, prolactin inhibitory factors; ADNI, Alzheimer’s Disease Neuroimaging Initiative; CSF, Cerebrospinal Fluid; eQTL, expression quantitative trait locus; Knight ADRC, Knight-Alzheimer’s Disease Research Center at Washington University School of Medicine; SNP, single nucleotide polymorphism; UTRs, untranslated regions.

## Additional files

Additional file 1: File contains a table of SNPs significantly associated with prolactin levels in blood plasma by meta-analysis. (DOCX 130 kb) Additional file 2: File contains a table of SNPs significantly associated with prolactin levels in CSF by meta-analysis. (DOCX 183 kb) Additional file 3: File contains a Q-Q plot of the plasma data used in this study. (DOCX 74 kb) Additional file 4: File contains a Q-Q plot of the CSF data used in this study. (DOCX 76 kb)
