# Supplementary material for: Genome-wide association study of prolactin levels in blood plasma and cerebrospinal fluid
Source: BMC Genomics. 2016 Jun 29;17(Suppl 3):436. doi: 10.1186/s12864-016-2785-0 (PMC4943503; doi:10.1186/s12864-016-2785-0)
Supplement: Additional file 1: — File contains a table of SNPs significantly associated with prolactin levels in blood plasma by meta-analysis. (DOCX 130 kb) [file 12864_2016_2785_MOESM1_ESM.docx]

| **SNP** | **CHR** | **Chromosomal position** | | **Proximal Gene(s)** | **MAF** | **Predicted Function** | **Meta-analysis**  **p-value** | |  |
| --- | --- | --- | --- | --- | --- | --- | --- | --- | --- |
| rs4512596 | 1 | 80708585 | ELTD1,LOC101927412 | | NA | intergenic | | 1.65E-08 | |
| rs10874121 | 1 | 80708882 | ELTD1,LOC101927412 | | NA | intergenic | | 1.65E-08 | |
| rs1342925 | 1 | 80709060 | ELTD1,LOC101927412 | | NA | intergenic | | 1.65E-08 | |
| rs1342924 | 1 | 80709156 | ELTD1,LOC101927412 | | NA | intergenic | | 1.65E-08 | |
| rs1342923 | 1 | 80709203 | ELTD1,LOC101927412 | | NA | intergenic | | 1.65E-08 | |
| rs80155001 | 1 | 81630896 | LOC101927412,LOC101927434 | | 0.092 | intergenic | | 5.08E-09 | |
| rs13408093 | 2 | 12871192 | TRIB2 | | 0.07 | intronic | | 6.88E-10 | |
| rs79045563 | 2 | 16964769 | FAM49A,RAD51AP2 | | 0.092 | intergenic | | 1.53E-09 | |
| rs74584815 | 2 | 16964812 | FAM49A,RAD51AP2 | | 0.1 | intergenic | | 1.53E-09 | |
| rs10496830 | 2 | 140666322 | YY1P2,LRP1B | | 0.11 | intergenic | | 1.10E-08 | |
| rs73963211 | 2 | 140669499 | YY1P2,LRP1B | | 0.11 | intergenic | | 1.10E-08 | |
| rs57605084 | 2 | 140672598 | YY1P2,LRP1B | | 0.11 | intergenic | | 1.10E-08 | |
| rs73836736 | 3 | 60438844 | FHIT | | 0.05 | intronic | | 1.16E-14 | |
| rs60612410 | 3 | 60439669 | FHIT | | 0.05 | intronic | | 1.16E-14 | |
| rs73833907 | 3 | 60448563 | FHIT | | 0.05 | intronic | | 1.18E-14 | |
| rs72878727 | 3 | 60448890 | FHIT | | 0.05 | intronic | | 1.18E-14 | |
| 4:80549170 | 4 | 80549170 | LINC00989,PCAT4 | | 0.045 | intergenic | | 5.32E-08 | |
| rs58152449 | 5 | 79243934 | CMYA5,MTX3 | | 0.12 | intergenic | | 1.06E-08 | |
| rs60907623 | 5 | 79243962 | CMYA5,MTX3 | | 0.12 | intergenic | | 9.04E-09 | |
| rs55726368 | 5 | 79245341 | CMYA5,MTX3 | | 0.12 | intergenic | | 1.04E-08 | |
| rs6871326 | 5 | 79246569 | CMYA5,MTX3 | | 0.12 | intergenic | | 9.98E-09 | |
| rs56228882 | 5 | 79249478 | CMYA5,MTX3 | | 0.11 | intergenic | | 1.68E-09 | |
| rs988084 | 6 | 28177492 | ZNF192P1,TOB2P1 | | NA | intergenic | | 3.28E-09 | |
| rs988083 | 6 | 28177588 | ZNF192P1,TOB2P1 | | NA | intergenic | | 3.28E-09 | |
| rs1150701 | 6 | 28183886 | TOB2P1 | | 0.14 | ncRNA_exonic | | 1.18E-08 | |
| rs1150702 | 6 | 28184097 | TOB2P1 | | 0.14 | ncRNA_exonic | | 1.18E-08 | |
| rs1150703 | 6 | 28184260 | TOB2P1 | | NA | ncRNA_exonic | | 3.28E-09 | |
| rs1233712 | 6 | 28193131 | ZSCAN9 | | 0.14 | UTR5 | | 1.18E-08 | |
| rs73726888 | 7 | 150218117 | GIMAP7 | | 0.089 | UTR3 | | 4.21E-09 | |
| rs10283305 | 8 | 14575970 | SGCZ | | 0.069 | intronic | | 8.67E-09 | |
| rs75139979 | 8 | 14579813 | SGCZ | | 0.068 | intronic | | 8.67E-09 | |
| rs76079150 | 8 | 14579969 | SGCZ | | 0.069 | intronic | | 8.92E-09 | |
| rs12548348 | 8 | 70430077 | SULF1 | | 0.12 | intronic | | 6.29E-11 | |
| rs5742734 | 14 | 23057001 | DAD1 | | 0.045 | intronic | | 5.14E-08 | |
| rs8073041 | 17 | 47498253 | PHB,LOC101927207 | | 0.073 | intergenic | | 7.87E-09 | |
| rs79268972 | 17 | 47531241 | PHB,LOC101927207 | | 0.075 | intergenic | | 2.55E-08 | |
| rs77482998 | 17 | 47532356 | PHB,LOC101927207 | | 0.067 | intergenic | | 4.61E-08 | |
