# Supplementary material for: Genome-wide association study of prolactin levels in blood plasma and cerebrospinal fluid
Source: BMC Genomics. 2016 Jun 29;17(Suppl 3):436. doi: 10.1186/s12864-016-2785-0 (PMC4943503; doi:10.1186/s12864-016-2785-0)
Supplement: Additional file 2: — File contains a table of SNPs significantly associated with prolactin levels in CSF by meta-analysis. (DOCX 183 kb) [file 12864_2016_2785_MOESM2_ESM.docx]

| **SNP** | **CHR** | **Chromosomal position** | **Proximal Gene(s)** | **MAF** | | **Predicted Function** | | **Meta-analysis**  **p-value** | |
| --- | --- | --- | --- | --- | --- | --- | --- | --- | --- |
| rs11589241 | 1 | 63498853 | ATG4C,LINC00466 | | 0.11 | | intergenic | | 5.74E-08 |
| rs116599282 | 1 | 81594752 | LOC101927412,LOC101927434 | | 0.024 | | intergenic | | 7.67E-11 |
| rs114085347 | 1 | 111007434 | PROK1,CYMP | | 0.037 | | intergenic | | 1.61E-11 |
| rs74722164 | 1 | 164733974 | PBX1 | | 0.009 | | intronic | | 8.76E-09 |
| rs59192532 | 1 | 170486444 | LOC101928650 | | 0.074 | | ncRNA_intronic | | 1.68E-14 |
| rs328633 | 2 | 9246648 | MBOAT2,ASAP2 | |  | | intergenic | | 4.07E-25 |
| rs1027958 | 2 | 9257720 | MBOAT2,ASAP2 | |  | | intergenic | | 3.81E-25 |
| rs4405730 | 2 | 9258229 | MBOAT2,ASAP2 | |  | | intergenic | | 3.81E-25 |
| rs1397497 | 2 | 9263035 | MBOAT2,ASAP2 | |  | | intergenic | | 3.81E-25 |
| rs6724103 | 2 | 9484542 | ASAP2 | |  | | intronic | | 9.57E-22 |
| rs10206519 | 2 | 9486582 | ASAP2 | |  | | intronic | | 1.91E-23 |
| rs4294977 | 2 | 9487069 | ASAP2 | |  | | intronic | | 1.91E-23 |
| rs10929573 | 2 | 9487249 | ASAP2 | |  | | intronic | | 1.91E-23 |
| rs7586595 | 2 | 9488245 | ASAP2 | |  | | intronic | | 1.91E-23 |
| rs4668617 | 2 | 9488656 | ASAP2 | |  | | intronic | | 1.91E-23 |
| rs4669384 | 2 | 9489022 | ASAP2 | |  | | intronic | | 1.91E-23 |
| rs4669385 | 2 | 9491017 | ASAP2 | |  | | exonic | | 9.57E-22 |
| rs7600712 | 2 | 9491586 | ASAP2 | |  | | intronic | | 9.57E-22 |
| rs7564293 | 2 | 9491669 | ASAP2 | |  | | intronic | | 1.91E-23 |
| rs4508567 | 2 | 9493297 | ASAP2 | | 0.12 | | intronic | | 9.57E-22 |
| rs2715863 | 2 | 9493546 | ASAP2 | | 0.12 | | intronic | | 9.57E-22 |
| rs2715865 | 2 | 9494190 | ASAP2 | |  | | intronic | | 9.57E-22 |
| rs57325322 | 2 | 9498398 | ASAP2 | | 0.13 | | intronic | | 9.57E-22 |
| rs59670261 | 2 | 9498766 | ASAP2 | | 0.13 | | intronic | | 9.57E-22 |
| rs2709574 | 2 | 9501735 | ASAP2 | | 0.11 | | intronic | | 8.48E-21 |
| rs2715870 | 2 | 9503326 | ASAP2 | | 0.14 | | intronic | | 8.48E-21 |
| rs2709573 | 2 | 9503816 | ASAP2 | | 0.14 | | intronic | | 2.26E-19 |
| rs2715872 | 2 | 9505187 | ASAP2 | | 0.14 | | intronic | | 8.48E-21 |
| rs2709572 | 2 | 9506167 | ASAP2 | | 0.14 | | intronic | | 8.48E-21 |
| rs2709571 | 2 | 9507133 | ASAP2 | | 0.12 | | intronic | | 8.76E-21 |
| rs2715874 | 2 | 9507413 | ASAP2 | | 0.12 | | intronic | | 8.76E-21 |
| rs2709578 | 2 | 9508908 | ASAP2 | | 0.11 | | intronic | | 8.76E-21 |
| rs2715877 | 2 | 9510783 | ASAP2 | | 0.11 | | intronic | | 8.76E-21 |
| rs13406297 | 2 | 9892227 | YWHAQ,TAF1B | | 0.081 | | intergenic | | 1.02E-24 |
| rs10175265 | 2 | 9892690 | YWHAQ,TAF1B | | 0.08 | | intergenic | | 1.02E-24 |
| rs10198218 | 2 | 9892763 | YWHAQ,TAF1B | | 0.089 | | intergenic | | 1.02E-24 |
| rs1652684 | 2 | 10010300 | TAF1B | |  | | intronic | | 7.57E-25 |
| rs406546 | 2 | 10010590 | TAF1B | |  | | intronic | | 7.57E-25 |
| rs394952 | 2 | 10011234 | TAF1B | |  | | intronic | | 9.07E-25 |
| rs396416 | 2 | 10013070 | TAF1B | |  | | intronic | | 7.57E-25 |
| rs366852 | 2 | 10026741 | TAF1B | |  | | intronic | | 7.57E-25 |
| rs400098 | 2 | 10038736 | TAF1B | |  | | intronic | | 7.57E-25 |
| rs400362 | 2 | 10042449 | TAF1B | |  | | intronic | | 7.57E-25 |
| rs58632829 | 2 | 11706478 | GREB1 | | 0.15 | | intronic | | 1.19E-10 |
| rs10174816 | 2 | 11753955 | GREB1 | | 0.14 | | intronic | | 1.19E-10 |
| rs896873 | 2 | 12489326 | LOC100506457 | |  | | ncRNA_intronic | | 4.65E-11 |
| rs4668756 | 2 | 12490731 | LOC100506457 | |  | | ncRNA_intronic | | 6.57E-11 |
| rs13408093 | 2 | 12871192 | TRIB2 | | 0.07 | | intronic | | 2.12E-25 |
| rs6751444 | 2 | 15109489 | LOC653602,NBAS | | 0.12 | | intergenic | | 5.41E-08 |
| rs59887342 | 2 | 15110458 | LOC653602,NBAS | | 0.12 | | intergenic | | 5.41E-08 |
| rs16862196 | 2 | 15110711 | LOC653602,NBAS | | 0.12 | | intergenic | | 4.49E-21 |
| rs4670052 | 2 | 15111982 | LOC653602,NBAS | | 0.12 | | intergenic | | 4.49E-21 |
| rs10192826 | 2 | 15939624 | LOC101926966,MYCNUT | | 0.17 | | intergenic | | 8.55E-23 |
| rs1147109 | 2 | 20206899 | MATN3 | |  | | intronic | | 1.61E-08 |
| rs56024303 | 2 | 75797267 | EVA1A | | 0.047 | | upstream | | 6.80E-09 |
| rs10209738 | 2 | 75797847 | EVA1A | | 0.08 | | upstream | | 6.81E-09 |
| rs10181984 | 2 | 75803357 | EVA1A,MRPL19 | | 0.075 | | intergenic | | 6.81E-09 |
| 2:173057317 | 2 | 173057317 | DLX2-AS1,ITGA6 | | 0.011 | | intergenic | | 2.54E-08 |
| rs79136631 | 3 | 3554379 | CRBN,LRRN1 | | 0.051 | | intergenic | | 1.06E-09 |
| rs59299638 | 3 | 3555230 | CRBN,LRRN1 | | 0.064 | | intergenic | | 1.07E-09 |
| rs60006665 | 3 | 3555425 | CRBN,LRRN1 | | 0.064 | | intergenic | | 1.07E-09 |
| rs73808148 | 3 | 3556002 | CRBN,LRRN1 | | 0.064 | | intergenic | | 1.07E-09 |
| 3:139332564 | 3 | 139332564 | NMNAT3 | | 0.0068 | | intronic | | 2.66E-08 |
| rs6776801 | 3 | 149939998 | LOC646903,LINC01213 | | 0.1 | | intergenic | | 1.88E-08 |
| rs75351074 | 3 | 162308071 | OTOL1,LINC01192 | | 0.072 | | intergenic | | 1.60E-09 |
| rs6781502 | 3 | 192650971 | MB21D2,HRASLS | | 0.042 | | intergenic | | 5.24E-08 |
| rs116209385 | 3 | 192655038 | MB21D2,HRASLS | | 0.042 | | intergenic | | 5.60E-08 |
| rs7609857 | 3 | 192661510 | MB21D2,HRASLS | | 0.053 | | intergenic | | 5.60E-08 |
| rs79958735 | 3 | 192663286 | MB21D2,HRASLS | | 0.053 | | intergenic | | 5.60E-08 |
| rs7616551 | 3 | 192667022 | MB21D2,HRASLS | | 0.048 | | intergenic | | 5.60E-08 |
| rs77191540 | 3 | 192669283 | MB21D2,HRASLS | | 0.048 | | intergenic | | 5.60E-08 |
| rs78437314 | 3 | 192669442 | MB21D2,HRASLS | | 0.053 | | intergenic | | 5.60E-08 |
| rs77290913 | 3 | 192669772 | MB21D2,HRASLS | | 0.048 | | intergenic | | 5.60E-08 |
| rs111390139 | 3 | 192670641 | MB21D2,HRASLS | | 0.053 | | intergenic | | 5.60E-08 |
| rs111479881 | 3 | 192670681 | MB21D2,HRASLS | | 0.054 | | intergenic | | 5.60E-08 |
| rs7634296 | 3 | 192670905 | MB21D2,HRASLS | | 0.053 | | intergenic | | 5.60E-08 |
| rs115323506 | 3 | 192672119 | MB21D2,HRASLS | | 0.053 | | intergenic | | 5.60E-08 |
| rs76706199 | 3 | 192672488 | MB21D2,HRASLS | | 0.053 | | intergenic | | 5.60E-08 |
| rs79511022 | 3 | 192674982 | MB21D2,HRASLS | | 0.048 | | intergenic | | 5.60E-08 |
| 3:192679339 | 3 | 192679339 | MB21D2,HRASLS | | 0.048 | | intergenic | | 5.24E-08 |
| 3:192682818 | 3 | 192682818 | MB21D2,HRASLS | | 0.048 | | intergenic | | 5.24E-08 |
| 3:192685098 | 3 | 192685098 | MB21D2,HRASLS | | 0.053 | | intergenic | | 5.24E-08 |
| rs6444677 | 3 | 192701646 | MB21D2,HRASLS | | 0.11 | | intergenic | | 9.10E-09 |
| rs6771012 | 3 | 192703868 | MB21D2,HRASLS | | 0.11 | | intergenic | | 9.10E-09 |
| rs73888035 | 3 | 192705136 | MB21D2,HRASLS | | 0.11 | | intergenic | | 9.10E-09 |
| rs73888036 | 3 | 192705704 | MB21D2,HRASLS | | 0.11 | | intergenic | | 9.10E-09 |
| rs6444678 | 3 | 192706858 | MB21D2,HRASLS | | 0.11 | | intergenic | | 9.10E-09 |
| rs73888039 | 3 | 192707795 | MB21D2,HRASLS | | 0.1 | | intergenic | | 9.10E-09 |
| rs7623151 | 3 | 192710881 | MB21D2,HRASLS | | 0.1 | | intergenic | | 9.10E-09 |
| rs73888044 | 3 | 192711958 | MB21D2,HRASLS | | 0.1 | | intergenic | | 9.10E-09 |
| rs12635543 | 3 | 192712332 | MB21D2,HRASLS | | 0.1 | | intergenic | | 9.10E-09 |
| rs73888045 | 3 | 192712352 | MB21D2,HRASLS | | 0.1 | | intergenic | | 9.10E-09 |
| 3:192714069 | 3 | 192714069 | MB21D2,HRASLS | | 0.1 | | intergenic | | 8.67E-09 |
| rs113447535 | 3 | 192714536 | MB21D2,HRASLS | | 0.1 | | intergenic | | 7.60E-09 |
| 3:192717002 | 3 | 192717002 | MB21D2,HRASLS | | 0.1 | | intergenic | | 8.67E-09 |
| 3:192717054 | 3 | 192717054 | MB21D2,HRASLS | | 0.14 | | intergenic | | 1.90E-09 |
| 3:192717126 | 3 | 192717126 | MB21D2,HRASLS | | 0.1 | | intergenic | | 8.67E-09 |
| rs57485637 | 3 | 192717255 | MB21D2,HRASLS | | 0.1 | | intergenic | | 8.67E-09 |
| rs56240096 | 3 | 192727729 | MB21D2,HRASLS | | 0.1 | | intergenic | | 9.10E-09 |
| rs113390842 | 3 | 192736088 | MB21D2,HRASLS | | 0.1 | | intergenic | | 9.10E-09 |
| rs57858478 | 4 | 6427210 | PPP2R2C | | 0.048 | | intronic | | 6.76E-10 |
| 4:73779311 | 4 | 73779311 | ADAMTS3,COX18 | | 0.037 | | intergenic | | 4.38E-09 |
| 4:73779312 | 4 | 73779312 | ADAMTS3,COX18 | | 0.037 | | intergenic | | 4.38E-09 |
| rs12510724 | 4 | 73786122 | ADAMTS3,COX18 | | 0.049 | | intergenic | | 4.41E-09 |
| rs78293930 | 4 | 73789543 | ADAMTS3,COX18 | | 0.049 | | intergenic | | 4.41E-09 |
| rs77877066 | 4 | 73792852 | ADAMTS3,COX18 | | 0.057 | | intergenic | | 1.11E-08 |
| 4:73794866 | 4 | 73794866 | ADAMTS3,COX18 | | 0.049 | | intergenic | | 4.41E-09 |
| rs116400506 | 4 | 73803343 | ADAMTS3,COX18 | | 0.049 | | intergenic | | 4.41E-09 |
| rs79669236 | 4 | 73804311 | ADAMTS3,COX18 | | 0.05 | | intergenic | | 4.41E-09 |
| rs74567446 | 4 | 73805603 | ADAMTS3,COX18 | | 0.0064 | | intergenic | | 4.38E-09 |
| rs16848973 | 4 | 73810317 | ADAMTS3,COX18 | | 0.039 | | intergenic | | 4.63E-09 |
| rs75807177 | 4 | 73819394 | ADAMTS3,COX18 | | 0.061 | | intergenic | | 4.63E-09 |
| rs114596431 | 4 | 73821308 | ADAMTS3,COX18 | | 0.059 | | intergenic | | 4.63E-09 |
| 4:73822294 | 4 | 73822294 | ADAMTS3,COX18 | | 0.06 | | intergenic | | 4.63E-09 |
| rs16848995 | 4 | 73823428 | ADAMTS3,COX18 | | 0.06 | | intergenic | | 4.63E-09 |
| rs78705547 | 4 | 73825739 | ADAMTS3,COX18 | | 0.06 | | intergenic | | 4.63E-09 |
| rs2114150 | 4 | 73827122 | ADAMTS3,COX18 | | 0.06 | | intergenic | | 4.63E-09 |
| rs2114149 | 4 | 73827168 | ADAMTS3,COX18 | | 0.046 | | intergenic | | 4.63E-09 |
| rs78824120 | 4 | 73827261 | ADAMTS3,COX18 | | 0.036 | | intergenic | | 4.38E-09 |
| rs2162206 | 4 | 73827342 | ADAMTS3,COX18 | | 0.061 | | intergenic | | 4.63E-09 |
| rs76737514 | 4 | 73827406 | ADAMTS3,COX18 | | 0.06 | | intergenic | | 4.63E-09 |
| rs78056196 | 4 | 73827682 | ADAMTS3,COX18 | | 0.06 | | intergenic | | 4.63E-09 |
| rs76103205 | 4 | 73829927 | ADAMTS3,COX18 | | 0.06 | | intergenic | | 4.63E-09 |
| rs76867420 | 4 | 73830275 | ADAMTS3,COX18 | | 0.059 | | intergenic | | 4.63E-09 |
| rs12507585 | 4 | 73833430 | ADAMTS3,COX18 | | 0.053 | | intergenic | | 4.44E-09 |
| rs313943 | 4 | 113973766 | ANK2 | | 0.11 | | intronic | | 8.88E-11 |
| rs313947 | 4 | 113976844 | ANK2 | | 0.076 | | intronic | | 3.10E-09 |
| rs79170366 | 4 | 136645476 | PABPC4L,LINC00613 | | 0.022 | | intergenic | | 2.87E-10 |
| rs114145185 | 4 | 136656531 | PABPC4L,LINC00613 | | 0.022 | | intergenic | | 2.87E-10 |
| rs74679954 | 4 | 136684008 | PABPC4L,LINC00613 | | 0.031 | | intergenic | | 3.45E-10 |
| rs116169053 | 4 | 136695940 | PABPC4L,LINC00613 | | 0.025 | | intergenic | | 3.45E-10 |
| 4:144195971 | 4 | 144195971 | USP38,GAB1 | | 0.0084 | | intergenic | | 5.05E-08 |
| rs114442154 | 4 | 144214303 | USP38,GAB1 | | 0.0086 | | intergenic | | 5.05E-08 |
| 5:108818817 | 5 | 108818817 | PJA2,MAN2A1 | | 0.016 | | intergenic | | 1.52E-08 |
| rs17422574 | 5 | 109122180 | MAN2A1 | | 0.008 | | intronic | | 6.06E-09 |
| rs17423051 | 5 | 109128835 | MAN2A1 | | 0.008 | | intronic | | 6.06E-09 |
| rs17488820 | 5 | 109129888 | MAN2A1 | | 0.006 | | intronic | | 6.06E-09 |
| 5:150345094 | 5 | 150345094 | ZNF300P1,GPX3 | | 0.051 | | intergenic | | 3.25E-11 |
| 5:150347571 | 5 | 150347571 | ZNF300P1,GPX3 | | 0.051 | | intergenic | | 3.25E-11 |
| 5:150347776 | 5 | 150347776 | ZNF300P1,GPX3 | | 0.051 | | intergenic | | 3.25E-11 |
| 5:150348102 | 5 | 150348102 | ZNF300P1,GPX3 | | 0.051 | | intergenic | | 3.25E-11 |
| 5:150348730 | 5 | 150348730 | ZNF300P1,GPX3 | | 0.051 | | intergenic | | 3.25E-11 |
| rs77308675 | 5 | 150351014 | ZNF300P1,GPX3 | | 0.051 | | intergenic | | 3.25E-11 |
| rs12654027 | 5 | 150354640 | ZNF300P1,GPX3 | | 0.055 | | intergenic | | 3.33E-11 |
| rs79924405 | 5 | 150356810 | ZNF300P1,GPX3 | | 0.057 | | intergenic | | 3.33E-11 |
| rs75296938 | 5 | 150358248 | ZNF300P1,GPX3 | | 0.057 | | intergenic | | 3.33E-11 |
| rs75244902 | 5 | 150359910 | ZNF300P1,GPX3 | | 0.063 | | intergenic | | 3.33E-11 |
| rs78843150 | 5 | 150360886 | ZNF300P1,GPX3 | | 0.063 | | intergenic | | 3.33E-11 |
| rs77837245 | 5 | 150368369 | ZNF300P1,GPX3 | | 0.057 | | intergenic | | 3.25E-11 |
| rs10036847 | 5 | 150370785 | ZNF300P1,GPX3 | | 0.11 | | intergenic | | 1.94E-08 |
| 5:150370843 | 5 | 150370843 | ZNF300P1,GPX3 | | 0.047 | | intergenic | | 3.25E-11 |
| rs116024283 | 5 | 150372073 | ZNF300P1,GPX3 | | 0.047 | | intergenic | | 3.25E-11 |
| rs77654994 | 5 | 150372167 | ZNF300P1,GPX3 | | 0.047 | | intergenic | | 3.25E-11 |
| rs9637871 | 5 | 150382402 | ZNF300P1,GPX3 | | 0.05 | | intergenic | | 1.73E-08 |
| 5:150383651 | 5 | 150383651 | ZNF300P1,GPX3 | | 0.047 | | intergenic | | 3.25E-11 |
| rs11953386 | 5 | 150386713 | ZNF300P1,GPX3 | | 0.045 | | intergenic | | 3.27E-11 |
| rs11953395 | 5 | 150386887 | ZNF300P1,GPX3 | | 0.045 | | intergenic | | 3.25E-11 |
| rs11953398 | 5 | 150386907 | ZNF300P1,GPX3 | | 0.045 | | intergenic | | 3.25E-11 |
| rs79729372 | 5 | 150388901 | ZNF300P1,GPX3 | | 0.043 | | intergenic | | 1.20E-12 |
| rs73722209 | 6 | 7449813 | RIOK1,DSP | | 0.045 | | intergenic | | 4.46E-10 |
| rs988084 | 6 | 28177492 | ZNF192P1,TOB2P1 | |  | | intergenic | | 7.01E-26 |
| rs988083 | 6 | 28177588 | ZNF192P1,TOB2P1 | |  | | intergenic | | 7.01E-26 |
| rs1150701 | 6 | 28183886 | TOB2P1 | | 0.14 | | ncRNA_exonic | | 1.44E-26 |
| rs1150702 | 6 | 28184097 | TOB2P1 | | 0.14 | | ncRNA_exonic | | 1.44E-26 |
| rs1150703 | 6 | 28184260 | TOB2P1 | |  | | ncRNA_exonic | | 7.01E-26 |
| rs1233712 | 6 | 28193131 | ZSCAN9 | | 0.14 | | UTR5 | | 1.44E-26 |
| rs1233706 | 6 | 28205711 | ZSCAN9,ZKSCAN4 | | . | | intergenic | | 4.63E-26 |
| rs1150708 | 6 | 28205827 | ZSCAN9,ZKSCAN4 | | 0.13 | | intergenic | | 4.63E-26 |
| 6:68512342 | 6 | 68512342 | SLC25A51P1,LOC102723883 | | . | | intergenic | | 1.26E-08 |
| rs115954659 | 6 | 164106967 | QKI,C6orf118 | | 0.024 | | intergenic | | 1.31E-24 |
| rs79421657 | 6 | 164135647 | QKI,C6orf118 | | 0.039 | | intergenic | | 1.26E-24 |
| rs115376916 | 6 | 164147638 | QKI,C6orf118 | | 0.037 | | intergenic | | 1.26E-24 |
| rs73726888 | 7 | 150218117 | GIMAP7 | | 0.089 | | UTR3 | | 6.44E-24 |
| rs16879110 | 8 | 32140742 | NRG1 | | 0.047 | | intronic | | 1.48E-08 |
| rs16879112 | 8 | 32141047 | NRG1 | | 0.047 | | intronic | | 1.48E-08 |
| rs77828259 | 8 | 32142785 | NRG1 | | 0.046 | | intronic | | 1.54E-08 |
| rs34940183 | 8 | 69411142 | C8orf34 | | 0.12 | | intronic | | 5.04E-09 |
| rs34428583 | 8 | 69411426 | C8orf34 | | 0.12 | | intronic | | 5.04E-09 |
| rs7000143 | 8 | 69411891 | C8orf34 | | 0.12 | | intronic | | 5.04E-09 |
| rs12548348 | 8 | 70430077 | SULF1 | | 0.12 | | intronic | | 9.84E-26 |
| rs12682456 | 8 | 70441579 | SULF1 | | 0.16 | | intronic | | 2.25E-08 |
| rs12680297 | 8 | 70441622 | SULF1 | | 0.15 | | intronic | | 2.25E-08 |
| rs12680331 | 8 | 70441834 | SULF1 | |  | | intronic | | 2.25E-08 |
| rs16936012 | 8 | 70442873 | SULF1 | |  | | intronic | | 2.25E-08 |
| rs16936014 | 8 | 70443081 | SULF1 | | 0.14 | | intronic | | 1.59E-11 |
| rs16936015 | 8 | 70443296 | SULF1 | | 0.15 | | intronic | | 2.25E-08 |
| rs16936017 | 8 | 70443544 | SULF1 | | 0.16 | | intronic | | 2.25E-08 |
| rs16936018 | 8 | 70444050 | SULF1 | | 0.16 | | intronic | | 2.25E-08 |
| rs16936019 | 8 | 70444480 | SULF1 | | 0.17 | | intronic | | 2.25E-08 |
| rs4737982 | 8 | 70445236 | SULF1 | | 0.15 | | intronic | | 2.25E-08 |
| rs10957495 | 8 | 70445363 | SULF1 | |  | | intronic | | 2.25E-08 |
| rs12674484 | 8 | 70445825 | SULF1 | | 0.17 | | intronic | | 2.25E-08 |
| rs77813747 | 8 | 70445882 | SULF1 | | 0.17 | | intronic | | 2.25E-08 |
| rs75158001 | 8 | 70445963 | SULF1 | | 0.17 | | intronic | | 2.25E-08 |
| rs16936020 | 8 | 70446086 | SULF1 | | 0.17 | | intronic | | 2.25E-08 |
| rs16936022 | 8 | 70446185 | SULF1 | | 0.17 | | intronic | | 2.25E-08 |
| rs4737984 | 8 | 70446533 | SULF1 | | 0.17 | | intronic | | 2.25E-08 |
| rs2197029 | 8 | 70447025 | SULF1 | | 0.18 | | intronic | | 2.25E-08 |
| rs2197030 | 8 | 70447058 | SULF1 | | 0.2 | | intronic | | 2.25E-08 |
| rs16936028 | 8 | 70447844 | SULF1 | | 0.19 | | intronic | | 2.25E-08 |
| rs16936031 | 8 | 70448627 | SULF1 | | 0.19 | | intronic | | 2.25E-08 |
| rs12541962 | 8 | 70448930 | SULF1 | |  | | intronic | | 2.25E-08 |
| rs73280513 | 8 | 87811577 | CNGB3,CNBD1 | | 0.04 | | intergenic | | 3.31E-14 |
| rs73280516 | 8 | 87811692 | CNGB3,CNBD1 | | 0.04 | | intergenic | | 3.31E-14 |
| rs11997198 | 8 | 87812547 | CNGB3,CNBD1 | | 0.039 | | intergenic | | 3.31E-14 |
| rs11987426 | 8 | 87812634 | CNGB3,CNBD1 | | 0.039 | | intergenic | | 3.31E-14 |
| rs11986670 | 8 | 87812712 | CNGB3,CNBD1 | | 0.039 | | intergenic | | 3.31E-14 |
| rs11987459 | 8 | 87812776 | CNGB3,CNBD1 | | 0.039 | | intergenic | | 3.31E-14 |
| rs11987570 | 8 | 87813298 | CNGB3,CNBD1 | | 0.04 | | intergenic | | 3.31E-14 |
| rs73280523 | 8 | 87813477 | CNGB3,CNBD1 | | 0.04 | | intergenic | | 3.31E-14 |
| rs11998218 | 8 | 87813492 | CNGB3,CNBD1 | | 0.04 | | intergenic | | 3.31E-14 |
| rs73263837 | 8 | 87846334 | CNGB3,CNBD1 | | 0.1 | | intergenic | | 8.23E-10 |
| rs113368559 | 8 | 87857000 | CNGB3,CNBD1 | | 0.1 | | intergenic | | 8.23E-10 |
| rs75685697 | 8 | 108809260 | ANGPT1,RSPO2 | | 0.023 | | intergenic | | 1.14E-08 |
| rs78143047 | 9 | 5527351 | PDCD1LG2 | | 0.055 | | intronic | | 3.61E-10 |
| rs114917525 | 9 | 17055600 | BNC2,CNTLN | | 0.02 | | intergenic | | 6.43E-09 |
| rs113992717 | 9 | 17125860 | BNC2,CNTLN | | 0.027 | | intergenic | | 5.60E-21 |
| rs16935348 | 9 | 17138777 | CNTLN | | 0.015 | | intronic | | 4.45E-21 |
| rs116566237 | 9 | 17147522 | CNTLN | | 0.016 | | intronic | | 3.70E-21 |
| 9:17150053 | 9 | 17150053 | CNTLN | | 0.016 | | intronic | | 4.53E-09 |
| 9:17150598 | 9 | 17150598 | CNTLN | | 0.016 | | intronic | | 4.53E-09 |
| 9:17150748 | 9 | 17150748 | CNTLN | | 0.016 | | intronic | | 4.53E-09 |
| 9:17150776 | 9 | 17150776 | CNTLN | | 0.016 | | intronic | | 4.53E-09 |
| 9:17150954 | 9 | 17150954 | CNTLN | | 0.016 | | intronic | | 4.53E-09 |
| rs34224729 | 9 | 17151356 | CNTLN | | 0.024 | | intronic | | 4.53E-09 |
| 9:17151586 | 9 | 17151586 | CNTLN | | 0.016 | | intronic | | 4.53E-09 |
| rs113899251 | 9 | 17151903 | CNTLN | | 0.016 | | intronic | | 4.53E-09 |
| 9:17151977 | 9 | 17151977 | CNTLN | | 0.016 | | intronic | | 4.53E-09 |
| 9:17152375 | 9 | 17152375 | CNTLN | | 0.016 | | intronic | | 4.53E-09 |
| 9:17152532 | 9 | 17152532 | CNTLN | | 0.016 | | intronic | | 4.53E-09 |
| rs114930128 | 9 | 17153518 | CNTLN | | 0.016 | | intronic | | 4.53E-09 |
| rs114397203 | 9 | 17154090 | CNTLN | | 0.016 | | intronic | | 4.53E-09 |
| rs115522943 | 9 | 17156130 | CNTLN | | 0.016 | | intronic | | 4.53E-09 |
| rs115948607 | 9 | 17158421 | CNTLN | | 0.014 | | intronic | | 4.53E-09 |
| 9:17158700 | 9 | 17158700 | CNTLN | | 0.015 | | intronic | | 4.53E-09 |
| rs7036370 | 9 | 17160053 | CNTLN | | 0.015 | | intronic | | 4.53E-09 |
| rs7020252 | 9 | 17160348 | CNTLN | | 0.015 | | intronic | | 4.53E-09 |
| rs59274061 | 9 | 17160818 | CNTLN | | 0.015 | | intronic | | 4.53E-09 |
| rs116054708 | 9 | 17161142 | CNTLN | | 0.015 | | intronic | | 4.53E-09 |
| rs7025329 | 9 | 17161682 | CNTLN | | 0.016 | | intronic | | 4.53E-09 |
| rs7025130 | 9 | 17161792 | CNTLN | | 0.015 | | intronic | | 4.53E-09 |
| rs7025248 | 9 | 17161857 | CNTLN | | 0.015 | | intronic | | 4.53E-09 |
| rs7029226 | 9 | 17162401 | CNTLN | | 0.015 | | intronic | | 4.53E-09 |
| rs16935377 | 9 | 17162572 | CNTLN | | 0.015 | | intronic | | 4.53E-09 |
| rs16935382 | 9 | 17162902 | CNTLN | | 0.015 | | intronic | | 4.53E-09 |
| rs114200181 | 9 | 17163174 | CNTLN | | 0.015 | | intronic | | 4.53E-09 |
| rs7847317 | 9 | 17163433 | CNTLN | | 0.015 | | intronic | | 4.53E-09 |
| rs7861471 | 9 | 17163894 | CNTLN | | 0.015 | | intronic | | 4.53E-09 |
| 9:17164417 | 9 | 17164417 | CNTLN | | 0.015 | | intronic | | 4.53E-09 |
| rs13286174 | 9 | 17164541 | CNTLN | | 0.015 | | intronic | | 4.53E-09 |
| rs115466020 | 9 | 17164542 | CNTLN | | 0.015 | | intronic | | 4.53E-09 |
| rs77998252 | 9 | 17165712 | CNTLN | | 0.015 | | intronic | | 4.53E-09 |
| rs116365570 | 9 | 17165836 | CNTLN | | 0.015 | | intronic | | 4.53E-09 |
| rs59700619 | 9 | 17166392 | CNTLN | | 0.015 | | intronic | | 4.53E-09 |
| rs61562344 | 9 | 17166593 | CNTLN | | 0.015 | | intronic | | 4.53E-09 |
| rs60503811 | 9 | 17166686 | CNTLN | | 0.015 | | intronic | | 4.53E-09 |
| rs57907512 | 9 | 17166843 | CNTLN | | 0.015 | | exonic | | 4.53E-09 |
| rs7037203 | 9 | 17167980 | CNTLN | | 0.015 | | UTR3 | | 4.53E-09 |
| rs7024284 | 9 | 17168380 | CNTLN | | 0.015 | | UTR3 | | 4.53E-09 |
| rs7856183 | 9 | 17169092 | CNTLN | | 0.015 | | intronic | | 4.53E-09 |
| rs7856549 | 9 | 17169424 | CNTLN | | 0.015 | | intronic | | 4.53E-09 |
| rs115328399 | 9 | 17169637 | CNTLN | | 0.015 | | intronic | | 4.53E-09 |
| rs116057383 | 9 | 17169655 | CNTLN | | 0.015 | | intronic | | 4.53E-09 |
| 9:17169889 | 9 | 17169889 | CNTLN | | 0.015 | | intronic | | 4.53E-09 |
| rs115545794 | 9 | 17169958 | CNTLN | | 0.015 | | intronic | | 4.53E-09 |
| 9:17170045 | 9 | 17170045 | CNTLN | | 0.015 | | intronic | | 4.53E-09 |
| rs116762987 | 9 | 17170103 | CNTLN | | 0.015 | | intronic | | 4.53E-09 |
| rs116085908 | 9 | 17170465 | CNTLN | | 0.015 | | intronic | | 4.53E-09 |
| rs114414695 | 9 | 17170515 | CNTLN | | 0.015 | | intronic | | 4.53E-09 |
| rs114075246 | 9 | 17170716 | CNTLN | | 0.015 | | intronic | | 4.53E-09 |
| rs114715742 | 9 | 17170806 | CNTLN | | 0.015 | | intronic | | 4.53E-09 |
| rs115773989 | 9 | 17171036 | CNTLN | | 0.015 | | intronic | | 4.53E-09 |
| rs116146287 | 9 | 17171147 | CNTLN | | 0.015 | | intronic | | 4.53E-09 |
| rs117565641 | 9 | 17171228 | CNTLN | | 0.016 | | intronic | | 4.53E-09 |
| rs79298739 | 9 | 17171300 | CNTLN | | 0.015 | | intronic | | 4.53E-09 |
| rs75566508 | 9 | 17171599 | CNTLN | | 0.015 | | intronic | | 4.53E-09 |
| rs60546162 | 9 | 17171759 | CNTLN | | 0.015 | | intronic | | 4.53E-09 |
| rs59698728 | 9 | 17172186 | CNTLN | | 0.015 | | intronic | | 4.53E-09 |
| 9:17172614 | 9 | 17172614 | CNTLN | | 0.015 | | intronic | | 4.53E-09 |
| 9:17172804 | 9 | 17172804 | CNTLN | | 0.015 | | intronic | | 4.53E-09 |
| rs116219658 | 9 | 17173180 | CNTLN | | 0.024 | | intronic | | 4.53E-09 |
| 9:17173248 | 9 | 17173248 | CNTLN | | 0.015 | | intronic | | 4.53E-09 |
| rs16935390 | 9 | 17173516 | CNTLN | | 0.015 | | intronic | | 4.53E-09 |
| rs58091700 | 9 | 17173842 | CNTLN | | 0.026 | | intronic | | 4.53E-09 |
| 9:17174504 | 9 | 17174504 | CNTLN | | 0.015 | | intronic | | 4.53E-09 |
| 9:17174618 | 9 | 17174618 | CNTLN | | 0.015 | | intronic | | 4.53E-09 |
| rs115452285 | 9 | 17176114 | CNTLN | | 0.015 | | intronic | | 4.53E-09 |
| 9:17177276 | 9 | 17177276 | CNTLN | | 0.015 | | intronic | | 4.53E-09 |
| 9:17177385 | 9 | 17177385 | CNTLN | | 0.015 | | intronic | | 4.53E-09 |
| rs111325437 | 9 | 17177401 | CNTLN | | 0.026 | | intronic | | 4.53E-09 |
| 9:17177956 | 9 | 17177956 | CNTLN | | 0.016 | | intronic | | 4.53E-09 |
| 9:17178271 | 9 | 17178271 | CNTLN | | 0.015 | | intronic | | 4.53E-09 |
| rs112015688 | 9 | 17178465 | CNTLN | | 0.03 | | intronic | | 4.53E-09 |
| 9:17178544 | 9 | 17178544 | CNTLN | | 0.015 | | intronic | | 4.53E-09 |
| rs114638020 | 9 | 17178546 | CNTLN | | 0.015 | | intronic | | 4.53E-09 |
| rs115432482 | 9 | 17178733 | CNTLN | | 0.015 | | intronic | | 4.53E-09 |
| 9:17178795 | 9 | 17178795 | CNTLN | | 0.015 | | intronic | | 4.53E-09 |
| rs114284753 | 9 | 17179313 | CNTLN | | 0.013 | | intronic | | 4.53E-09 |
| rs114941103 | 9 | 17179883 | CNTLN | | 0.015 | | intronic | | 4.53E-09 |
| rs60401056 | 9 | 17179982 | CNTLN | | 0.015 | | intronic | | 4.53E-09 |
| 9:17180524 | 9 | 17180524 | CNTLN | | 0.014 | | intronic | | 4.53E-09 |
| rs116802517 | 9 | 17181157 | CNTLN | | 0.014 | | intronic | | 4.53E-09 |
| rs114048778 | 9 | 17181300 | CNTLN | | 0.014 | | intronic | | 4.53E-09 |
| rs116403689 | 9 | 17181898 | CNTLN | | 0.015 | | intronic | | 4.53E-09 |
| rs115803412 | 9 | 17181947 | CNTLN | | 0.015 | | intronic | | 4.53E-09 |
| rs59305778 | 9 | 17183252 | CNTLN | | 0.014 | | intronic | | 4.53E-09 |
| rs111424898 | 9 | 17184442 | CNTLN | | 0.026 | | intronic | | 4.53E-09 |
| rs16935395 | 9 | 17184637 | CNTLN | | 0.022 | | intronic | | 4.53E-09 |
| rs111331091 | 9 | 17184700 | CNTLN | | 0.016 | | intronic | | 4.53E-09 |
| rs114602030 | 9 | 17185709 | CNTLN | | 0.015 | | intronic | | 4.53E-09 |
| rs16935397 | 9 | 17186374 | CNTLN | | 0.015 | | intronic | | 4.53E-09 |
| rs16935399 | 9 | 17187351 | CNTLN | | 0.015 | | intronic | | 4.53E-09 |
| rs115699726 | 9 | 17189889 | CNTLN | | 0.015 | | intronic | | 4.53E-09 |
| rs114710625 | 9 | 17190349 | CNTLN | | 0.015 | | intronic | | 4.53E-09 |
| 9:17190749 | 9 | 17190749 | CNTLN | | 0.015 | | intronic | | 4.53E-09 |
| rs112159564 | 9 | 17190904 | CNTLN | | 0.03 | | intronic | | 4.89E-09 |
| rs116052690 | 9 | 17192904 | CNTLN | | 0.018 | | intronic | | 4.53E-09 |
| 9:17193393 | 9 | 17193393 | CNTLN | | 0.014 | | intronic | | 4.53E-09 |
| rs114522694 | 9 | 17193566 | CNTLN | | 0.02 | | intronic | | 4.53E-09 |
| rs115177946 | 9 | 17193696 | CNTLN | | 0.018 | | intronic | | 4.53E-09 |
| 9:17193715 | 9 | 17193715 | CNTLN | | 0.018 | | intronic | | 4.53E-09 |
| rs59211421 | 9 | 17193719 | CNTLN | | 0.034 | | intronic | | 4.89E-09 |
| rs59453259 | 9 | 17193924 | CNTLN | | 0.018 | | intronic | | 4.53E-09 |
| rs59433459 | 9 | 17194300 | CNTLN | | 0.014 | | intronic | | 4.53E-09 |
| 9:17195024 | 9 | 17195024 | CNTLN | | 0.014 | | intronic | | 4.53E-09 |
| 9:17195765 | 9 | 17195765 | CNTLN | | 0.015 | | intronic | | 4.53E-09 |
| rs114991618 | 9 | 17196426 | CNTLN | | 0.018 | | intronic | | 4.53E-09 |
| rs115576261 | 9 | 17197096 | CNTLN | | 0.018 | | intronic | | 4.53E-09 |
| 9:17197155 | 9 | 17197155 | CNTLN | | 0.018 | | intronic | | 4.53E-09 |
| 9:17197990 | 9 | 17197990 | CNTLN | | 0.014 | | intronic | | 4.53E-09 |
| 9:17198122 | 9 | 17198122 | CNTLN | | 0.018 | | intronic | | 4.53E-09 |
| 9:17198738 | 9 | 17198738 | CNTLN | | 0.014 | | intronic | | 4.53E-09 |
| rs114391967 | 9 | 17199162 | CNTLN | | 0.018 | | intronic | | 4.53E-09 |
| 9:17199936 | 9 | 17199936 | CNTLN | | 0.027 | | intronic | | 4.81E-09 |
| 9:17200494 | 9 | 17200494 | CNTLN | | 0.015 | | intronic | | 4.53E-09 |
| 9:17201554 | 9 | 17201554 | CNTLN | | 0.014 | | intronic | | 4.53E-09 |
| rs114046801 | 9 | 17202157 | CNTLN | | 0.018 | | intronic | | 4.53E-09 |
| rs114696203 | 9 | 17202284 | CNTLN | | 0.018 | | intronic | | 4.53E-09 |
| rs114325006 | 9 | 17206278 | CNTLN | | 0.018 | | intronic | | 4.53E-09 |
| rs115570524 | 9 | 17206417 | CNTLN | | 0.018 | | intronic | | 4.53E-09 |
| rs116772452 | 9 | 17206701 | CNTLN | | 0.018 | | intronic | | 4.53E-09 |
| rs114630295 | 9 | 17207511 | CNTLN | | 0.018 | | intronic | | 4.53E-09 |
| rs114108034 | 9 | 17207549 | CNTLN | | 0.018 | | intronic | | 4.53E-09 |
| rs116362552 | 9 | 17207567 | CNTLN | | 0.029 | | intronic | | 4.53E-09 |
| rs116146554 | 9 | 17208265 | CNTLN | | 0.015 | | intronic | | 4.53E-09 |
| rs7847327 | 9 | 17209790 | CNTLN | | 0.018 | | intronic | | 4.53E-09 |
| 9:17210409 | 9 | 17210409 | CNTLN | | 0.018 | | intronic | | 4.53E-09 |
| 9:17210410 | 9 | 17210410 | CNTLN | | 0.018 | | intronic | | 4.53E-09 |
| rs112567153 | 9 | 17210740 | CNTLN | | 0.045 | | intronic | | 5.74E-09 |
| 9:17213660 | 9 | 17213660 | CNTLN | | 0.044 | | intronic | | 5.74E-09 |
| 9:17216585 | 9 | 17216585 | CNTLN | | 0.018 | | intronic | | 4.53E-09 |
| rs78460750 | 9 | 17217944 | CNTLN | | 0.044 | | intronic | | 5.74E-09 |
| rs111405738 | 9 | 17240952 | CNTLN | | 0.023 | | intronic | | 4.53E-09 |
| rs114769906 | 9 | 17246513 | CNTLN | | 0.022 | | intronic | | 4.53E-09 |
| 9:17295309 | 9 | 17295309 | CNTLN | | 0.011 | | intronic | | 4.07E-09 |
| rs78412979 | 9 | 17346680 | CNTLN | | 0.047 | | intronic | | 4.02E-09 |
| rs113595659 | 9 | 17347395 | CNTLN | | 0.047 | | intronic | | 4.07E-09 |
| rs113440396 | 9 | 17347733 | CNTLN | | 0.047 | | intronic | | 4.07E-09 |
| rs112516578 | 9 | 17347869 | CNTLN | | 0.047 | | intronic | | 4.07E-09 |
| rs112924446 | 9 | 17348344 | CNTLN | | 0.03 | | intronic | | 4.07E-09 |
| rs112659382 | 9 | 17348661 | CNTLN | | 0.03 | | intronic | | 4.07E-09 |
| rs111595408 | 9 | 17349976 | CNTLN | | 0.025 | | intronic | | 4.07E-09 |
| rs75931249 | 9 | 17352601 | CNTLN | | 0.026 | | intronic | | 4.07E-09 |
| rs113461813 | 9 | 17356381 | CNTLN | | 0.026 | | intronic | | 4.07E-09 |
| 9:17358746 | 9 | 17358746 | CNTLN | | 0.022 | | intronic | | 4.07E-09 |
| rs112582769 | 9 | 17376118 | CNTLN | | 0.013 | | intronic | | 4.07E-09 |
| rs113572060 | 9 | 17376254 | CNTLN | | 0.013 | | intronic | | 4.22E-09 |
| rs111930901 | 9 | 17378584 | CNTLN | | 0.016 | | intronic | | 4.22E-09 |
| rs11999572 | 9 | 17420411 | CNTLN | | 0.033 | | intronic | | 2.62E-09 |
| 9:17440448 | 9 | 17440448 | CNTLN | | 0.016 | | intronic | | 3.77E-09 |
| rs10812313 | 9 | 25870493 | LINC01241,LOC100506422 | | 0.018 | | intergenic | | 1.34E-09 |
| rs10967103 | 9 | 25872263 | LINC01241,LOC100506422 | | 0.018 | | intergenic | | 1.34E-09 |
| rs12004825 | 9 | 38159475 | SHB,ALDH1B1 | | 0.082 | | intergenic | | 9.24E-11 |
| rs4354401 | 9 | 38159692 | SHB,ALDH1B1 | | 0.083 | | intergenic | | 9.24E-11 |
| rs57446614 | 9 | 38161181 | SHB,ALDH1B1 | | 0.082 | | intergenic | | 9.24E-11 |
| rs1156779 | 9 | 38163095 | SHB,ALDH1B1 | |  | | intergenic | | 8.57E-11 |
| rs10814663 | 9 | 38163511 | SHB,ALDH1B1 | |  | | intergenic | | 1.39E-10 |
| rs73497003 | 9 | 101147171 | GABBR2 | | 0.092 | | intronic | | 8.24E-11 |
| rs10986085 | 9 | 101147811 | GABBR2 | | 0.092 | | intronic | | 8.24E-11 |
| rs7047634 | 9 | 101148876 | GABBR2 | | 0.092 | | intronic | | 8.24E-11 |
| rs75181764 | 9 | 101154814 | GABBR2 | | 0.056 | | intronic | | 9.60E-09 |
| rs16915579 | 9 | 101156399 | GABBR2 | | 0.054 | | intronic | | 1.18E-08 |
| rs78575005 | 10 | 31433274 | ZNF438,ZEB1-AS1 | | 0.096 | | intergenic | | 2.23E-12 |
| rs6537509 | 10 | 50428415 | C10orf128,C10orf71-AS1 | |  | | intergenic | | 1.37E-08 |
| rs7085254 | 10 | 50443038 | C10orf128,C10orf71-AS1 | |  | | intergenic | | 1.37E-08 |
| 10:61094181 | 10 | 61094181 | FAM13C | | 0.045 | | intronic | | 3.11E-08 |
| rs786935 | 10 | 102138903 | LINC00263 | |  | | ncRNA_intronic | | 9.59E-19 |
| 10:118361616 | 10 | 118361616 | PNLIPRP1 | | 0.043 | | intronic | | 2.37E-08 |
| rs7925141 | 11 | 4116725 | RRM1 | | 0.11 | | intronic | | 1.87E-24 |
| rs10836456 | 11 | 4697544 | OR51E1,OR51E2 | |  | | intergenic | | 1.81E-09 |
| rs111570535 | 11 | 5473509 | OR51B5 | | 0.088 | | intronic | | 4.76E-22 |
| rs11037506 | 11 | 5475759 | OR51B5 | | 0.083 | | intronic | | 4.76E-22 |
| rs10450568 | 11 | 5476449 | OR51B5 | | 0.085 | | intronic | | 7.23E-22 |
| rs77734342 | 11 | 5478191 | OR51B5 | | 0.061 | | intronic | | 6.65E-21 |
| rs12289566 | 11 | 5479480 | OR51B5 | | 0.062 | | intronic | | 6.65E-21 |
| rs12295862 | 11 | 5480193 | OR51B5 | | 0.062 | | intronic | | 6.65E-21 |
| rs4300398 | 11 | 8745055 | ST5 | |  | | intronic | | 3.17E-12 |
| rs10769946 | 11 | 8748490 | ST5 | |  | | intronic | | 2.13E-10 |
| rs6484423 | 11 | 8804111 | LOC102724784 | |  | | ncRNA_intronic | | 3.03E-12 |
| rs7127574 | 11 | 8807548 | LOC102724784 | |  | | ncRNA_intronic | | 3.03E-12 |
| rs11042083 | 11 | 8839725 | ST5 | |  | | intronic | | 1.83E-09 |
| rs4617587 | 11 | 8842015 | ST5 | |  | | intronic | | 1.83E-09 |
| rs7947427 | 11 | 8842486 | ST5 | |  | | intronic | | 1.83E-09 |
| rs4290228 | 11 | 8844087 | ST5 | |  | | intronic | | 1.83E-09 |
| rs7113023 | 11 | 8850225 | ST5 | |  | | intronic | | 1.83E-09 |
| rs2568050 | 11 | 8850704 | ST5 | |  | | intronic | | 1.83E-09 |
| rs2653589 | 11 | 8851208 | ST5 | |  | | intronic | | 1.93E-09 |
| rs2568071 | 11 | 8854309 | ST5 | |  | | intronic | | 1.83E-09 |
| rs2455604 | 11 | 8856822 | ST5 | |  | | intronic | | 1.83E-09 |
| rs2568028 | 11 | 8860688 | ST5 | |  | | intronic | | 1.83E-09 |
| rs2256067 | 11 | 8867303 | ST5 | |  | | intronic | | 1.83E-09 |
| rs2568051 | 11 | 8870245 | ST5 | |  | | intronic | | 1.83E-09 |
| rs2436178 | 11 | 8876581 | ST5 | |  | | intronic | | 1.83E-09 |
| rs2742551 | 11 | 8877052 | ST5 | |  | | intronic | | 1.83E-09 |
| rs2568055 | 11 | 8878803 | ST5 | |  | | intronic | | 1.83E-09 |
| rs2742548 | 11 | 8879663 | ST5 | |  | | intronic | | 1.83E-09 |
| rs2568058 | 11 | 8883112 | ST5 | |  | | intronic | | 1.83E-09 |
| rs2653614 | 11 | 8883968 | ST5 | |  | | intronic | | 1.83E-09 |
| rs2653615 | 11 | 8884472 | ST5 | |  | | intronic | | 1.83E-09 |
| rs2568060 | 11 | 8885155 | ST5 | |  | | intronic | | 1.83E-09 |
| rs2568063 | 11 | 8889283 | ST5 | |  | | intronic | | 1.83E-09 |
| rs2742543 | 11 | 8890569 | ST5 | |  | | intronic | | 1.83E-09 |
| rs2568041 | 11 | 8900358 | ST5 | |  | | intronic | | 1.29E-11 |
| rs2742539 | 11 | 8902214 | ST5 | |  | | intronic | | 1.29E-11 |
| rs7932678 | 11 | 8908817 | ST5 | |  | | intronic | | 1.29E-11 |
| rs10840143 | 11 | 8913217 | ST5 | |  | | intronic | | 1.41E-11 |
| rs2742530 | 11 | 8915367 | ST5 | |  | | intronic | | 3.35E-11 |
| rs9794991 | 11 | 8916404 | ST5 | |  | | intronic | | 1.37E-11 |
| rs2742528 | 11 | 8916953 | ST5 | |  | | intronic | | 2.35E-11 |
| rs2653563 | 11 | 8917737 | ST5 | |  | | intronic | | 1.26E-11 |
| rs2742527 | 11 | 8918154 | ST5 | |  | | intronic | | 1.26E-11 |
| rs2742526 | 11 | 8919872 | ST5 | |  | | intronic | | 1.26E-11 |
| rs2653561 | 11 | 8920150 | ST5 | |  | | intronic | | 1.26E-11 |
| rs2262323 | 11 | 8920576 | ST5 | |  | | intronic | | 1.26E-11 |
| rs2568036 | 11 | 8923778 | ST5 | |  | | intronic | | 1.26E-11 |
| rs2568033 | 11 | 8924552 | ST5 | |  | | intronic | | 2.56E-11 |
| rs2568030 | 11 | 8926205 | ST5 | |  | | intronic | | 1.26E-11 |
| rs2653583 | 11 | 8930157 | ST5 | |  | | intronic | | 1.26E-11 |
| rs2568027 | 11 | 8931093 | ST5 | |  | | intronic | | 1.26E-11 |
| rs2568026 | 11 | 8932733 | AKIP1 | |  | | UTR5 | | 1.29E-11 |
| rs2653579 | 11 | 8933789 | AKIP1 | |  | | intronic | | 1.26E-11 |
| rs2742518 | 11 | 8934101 | AKIP1 | |  | | intronic | | 1.26E-11 |
| rs3105215 | 11 | 8937392 | AKIP1 | |  | | intronic | | 1.26E-11 |
| rs2568024 | 11 | 8942426 | C11orf16 | |  | | intronic | | 1.26E-11 |
| rs2653608 | 11 | 9002401 | NRIP3 | |  | | UTR3 | | 8.12E-09 |
| rs2568046 | 11 | 9005036 | NRIP3 | |  | | UTR3 | | 8.12E-09 |
| rs2742469 | 11 | 9007651 | NRIP3 | |  | | intronic | | 8.12E-09 |
| rs6416158 | 11 | 9013584 | NRIP3 | |  | | intronic | | 8.12E-09 |
| rs1989143 | 11 | 9016746 | NRIP3 | |  | | intronic | | 8.12E-09 |
| rs7119887 | 11 | 9016935 | NRIP3 | |  | | intronic | | 8.12E-09 |
| rs7396427 | 11 | 9018199 | NRIP3 | |  | | intronic | | 8.12E-09 |
| rs7395934 | 11 | 9018292 | NRIP3 | |  | | intronic | | 8.12E-09 |
| rs7394940 | 11 | 9018702 | NRIP3 | |  | | intronic | | 8.12E-09 |
| rs7936297 | 11 | 9028126 | NRIP3,SCUBE2 | |  | | intergenic | | 8.12E-09 |
| rs7950826 | 11 | 9028924 | NRIP3,SCUBE2 | |  | | intergenic | | 8.12E-09 |
| rs7479205 | 11 | 9031023 | NRIP3,SCUBE2 | |  | | intergenic | | 7.07E-09 |
| rs10769982 | 11 | 9033786 | NRIP3,SCUBE2 | |  | | intergenic | | 5.35E-09 |
| rs7939313 | 11 | 9034289 | NRIP3,SCUBE2 | |  | | intergenic | | 5.62E-09 |
| rs6416159 | 11 | 9036308 | NRIP3,SCUBE2 | |  | | intergenic | | 5.48E-09 |
| rs1865557 | 11 | 9650624 | WEE1,SWAP70 | |  | | intergenic | | 3.17E-11 |
| rs2099465 | 11 | 9654703 | WEE1,SWAP70 | |  | | intergenic | | 3.17E-11 |
| rs3751001 | 11 | 9800821 | SBF2-AS1 | | 0.12 | | ncRNA_intronic | | 5.22E-08 |
| 11:11356777 | 11 | 11356777 | GALNT18 | | 0.012 | | intronic | | 1.93E-09 |
| rs115277988 | 11 | 94909052 | SESN3 | | 0.025 | | intronic | | 5.85E-10 |
| rs17621683 | 11 | 94917672 | SESN3 | | . | | exonic | | 5.85E-10 |
| rs74078832 | 12 | 41821610 | PDZRN4 | | 0.035 | | intronic | | 2.84E-08 |
| rs77882185 | 12 | 48318211 | VDR,TMEM106C | | 0.023 | | intergenic | | 7.93E-09 |
| rs76099334 | 12 | 48330420 | VDR,TMEM106C | | 0.059 | | intergenic | | 2.15E-09 |
| rs73361197 | 12 | 92254237 | DCN,C12orf79 | | 0.079 | | intergenic | | 4.11E-08 |
| rs11107133 | 12 | 94017839 | SOCS2,CRADD | | 0.22 | | intergenic | | 5.95E-08 |
| rs73373473 | 12 | 94018075 | SOCS2,CRADD | | 0.22 | | intergenic | | 5.95E-08 |
| rs80307508 | 12 | 94018105 | SOCS2,CRADD | | 0.22 | | intergenic | | 5.95E-08 |
| rs73373476 | 12 | 94018267 | SOCS2,CRADD | | 0.22 | | intergenic | | 5.95E-08 |
| rs111651314 | 12 | 94018291 | SOCS2,CRADD | | 0.22 | | intergenic | | 5.95E-08 |
| rs12299420 | 12 | 94085026 | CRADD | | . | | intronic | | 1.51E-12 |
| rs60773117 | 12 | 94141500 | CRADD | | 0.13 | | intronic | | 2.58E-09 |
| rs117015121 | 12 | 122236427 | LINC01089 | | 0.026 | | ncRNA_intronic | | 1.86E-11 |
| rs111566625 | 13 | 26829214 | CDK8 | | 0.058 | | intronic | | 4.09E-08 |
| rs9581678 | 13 | 27009647 | CDK8,WASF3 | | 0.034 | | intergenic | | 4.43E-09 |
| rs1410282 | 13 | 27009924 | CDK8,WASF3 | | 0.044 | | intergenic | | 2.31E-12 |
| rs4943591 | 13 | 39055128 | UFM1,LINC00437 | |  | | intergenic | | 1.01E-09 |
| rs5022766 | 13 | 39062367 | UFM1,LINC00437 | |  | | intergenic | | 1.01E-09 |
| rs2323918 | 13 | 39063238 | UFM1,LINC00437 | |  | | intergenic | | 1.01E-09 |
| rs2323919 | 13 | 39063247 | UFM1,LINC00437 | |  | | intergenic | | 1.01E-09 |
| rs2323921 | 13 | 39064687 | UFM1,LINC00437 | |  | | intergenic | | 1.01E-09 |
| rs2323922 | 13 | 39064717 | UFM1,LINC00437 | |  | | intergenic | | 1.01E-09 |
| rs2323923 | 13 | 39065024 | UFM1,LINC00437 | |  | | intergenic | | 1.01E-09 |
| rs4417431 | 13 | 39065057 | UFM1,LINC00437 | |  | | intergenic | | 1.01E-09 |
| rs2323924 | 13 | 39065321 | UFM1,LINC00437 | |  | | intergenic | | 1.01E-09 |
| rs4479105 | 13 | 39067258 | UFM1,LINC00437 | |  | | intergenic | | 1.01E-09 |
| rs7990453 | 13 | 39068334 | UFM1,LINC00437 | |  | | intergenic | | 1.03E-09 |
| rs7990016 | 13 | 39068503 | UFM1,LINC00437 | |  | | intergenic | | 1.03E-09 |
| rs4943592 | 13 | 39069009 | UFM1,LINC00437 | |  | | intergenic | | 1.03E-09 |
| rs4245389 | 13 | 39069197 | UFM1,LINC00437 | |  | | intergenic | | 1.03E-09 |
| rs9548371 | 13 | 39069842 | UFM1,LINC00437 | |  | | intergenic | | 1.03E-09 |
| rs78487207 | 13 | 39073563 | UFM1,LINC00437 | | 0.077 | | intergenic | | 1.03E-09 |
| rs2323927 | 13 | 39075408 | UFM1,LINC00437 | |  | | intergenic | | 1.11E-09 |
| rs11840889 | 13 | 39080466 | UFM1,LINC00437 | | 0.077 | | intergenic | | 1.03E-09 |
| rs75068514 | 13 | 39083876 | UFM1,LINC00437 | | 0.081 | | intergenic | | 1.03E-09 |
| rs77133214 | 13 | 67026297 | PCDH9 | | 0.025 | | intronic | | 1.90E-10 |
| rs79071582 | 13 | 67100497 | PCDH9 | | 0.023 | | intronic | | 3.37E-09 |
| 13:67108028 | 13 | 67108028 | PCDH9 | | 0.023 | | intronic | | 3.37E-09 |
| rs116953504 | 13 | 67119174 | PCDH9 | | 0.027 | | intronic | | 3.37E-09 |
| rs117456316 | 13 | 67136123 | PCDH9 | | 0.023 | | intronic | | 3.37E-09 |
| rs73513886 | 13 | 67139552 | PCDH9 | | 0.033 | | intronic | | 7.72E-10 |
| rs117893870 | 13 | 67144282 | PCDH9 | | 0.023 | | intronic | | 3.37E-09 |
| rs117796262 | 13 | 67147751 | PCDH9 | | 0.023 | | intronic | | 3.37E-09 |
| rs79847255 | 13 | 67152523 | PCDH9 | | 0.023 | | intronic | | 3.37E-09 |
| rs17081545 | 13 | 67165163 | PCDH9 | | 0.023 | | intronic | | 3.37E-09 |
| rs117145551 | 13 | 67166761 | PCDH9 | | 0.023 | | intronic | | 3.37E-09 |
| rs117328565 | 13 | 67171221 | PCDH9 | | 0.023 | | intronic | | 3.37E-09 |
| rs61700274 | 14 | 48694503 | LINC00648,RPS29 | | 0.091 | | intergenic | | 2.65E-10 |
| rs60816786 | 14 | 48694513 | LINC00648,RPS29 | | 0.091 | | intergenic | | 2.65E-10 |
| rs17119901 | 14 | 48700432 | LINC00648,RPS29 | | 0.089 | | intergenic | | 2.65E-10 |
| rs61170907 | 14 | 48701965 | LINC00648,RPS29 | | 0.089 | | intergenic | | 2.77E-10 |
| rs62009379 | 14 | 48702244 | LINC00648,RPS29 | | 0.092 | | intergenic | | 2.77E-10 |
| rs62009380 | 14 | 48704110 | LINC00648,RPS29 | | 0.089 | | intergenic | | 2.77E-10 |
| rs62009381 | 14 | 48706012 | LINC00648,RPS29 | | 0.089 | | intergenic | | 2.77E-10 |
| rs78879667 | 14 | 48706872 | LINC00648,RPS29 | | 0.087 | | intergenic | | 2.77E-10 |
| rs62009383 | 14 | 48712539 | LINC00648,RPS29 | | 0.09 | | intergenic | | 2.77E-10 |
| rs11622346 | 14 | 48713757 | LINC00648,RPS29 | | 0.085 | | intergenic | | 2.77E-10 |
| rs62009386 | 14 | 48718925 | LINC00648,RPS29 | | 0.088 | | intergenic | | 1.40E-09 |
| rs11621237 | 14 | 48721125 | LINC00648,RPS29 | | 0.086 | | intergenic | | 1.40E-09 |
| rs2416152 | 14 | 48723075 | LINC00648,RPS29 | | 0.09 | | intergenic | | 1.40E-09 |
| rs11620642 | 14 | 48724843 | LINC00648,RPS29 | | 0.086 | | intergenic | | 1.40E-09 |
| 14:48724919 | 14 | 48724919 | LINC00648,RPS29 | | 0.088 | | intergenic | | 1.40E-09 |
| rs11621629 | 14 | 48725332 | LINC00648,RPS29 | | 0.085 | | intergenic | | 1.40E-09 |
| 14:48726817 | 14 | 48726817 | LINC00648,RPS29 | | 0.089 | | intergenic | | 1.40E-09 |
| rs11157625 | 14 | 48727388 | LINC00648,RPS29 | | 0.086 | | intergenic | | 1.40E-09 |
| 14:48728139 | 14 | 48728139 | LINC00648,RPS29 | | 0.086 | | intergenic | | 1.40E-09 |
| rs11627319 | 14 | 48731583 | LINC00648,RPS29 | | 0.09 | | intergenic | | 1.40E-09 |
| rs11622860 | 14 | 48731932 | LINC00648,RPS29 | | 0.09 | | intergenic | | 1.40E-09 |
| rs11622862 | 14 | 48731955 | LINC00648,RPS29 | | 0.086 | | intergenic | | 1.40E-09 |
| rs11623786 | 14 | 48732141 | LINC00648,RPS29 | | 0.091 | | intergenic | | 1.40E-09 |
| rs11623754 | 14 | 48732173 | LINC00648,RPS29 | | 0.091 | | intergenic | | 1.40E-09 |
| rs36087410 | 14 | 48732454 | LINC00648,RPS29 | | 0.09 | | intergenic | | 1.40E-09 |
| 14:48732511 | 14 | 48732511 | LINC00648,RPS29 | | 0.09 | | intergenic | | 1.40E-09 |
| 14:48732560 | 14 | 48732560 | LINC00648,RPS29 | | 0.085 | | intergenic | | 1.40E-09 |
| rs111995703 | 14 | 48733614 | LINC00648,RPS29 | | 0.086 | | intergenic | | 1.40E-09 |
| rs113483719 | 14 | 48733758 | LINC00648,RPS29 | | 0.09 | | intergenic | | 1.40E-09 |
| rs62009429 | 14 | 48736528 | LINC00648,RPS29 | | 0.092 | | intergenic | | 1.40E-09 |
| rs72474148 | 14 | 48737264 | LINC00648,RPS29 | | 0.087 | | intergenic | | 1.40E-09 |
| rs62009435 | 14 | 48740224 | LINC00648,RPS29 | | 0.092 | | intergenic | | 1.40E-09 |
| rs62009436 | 14 | 48740635 | LINC00648,RPS29 | | 0.092 | | intergenic | | 1.40E-09 |
| rs61599120 | 14 | 48742787 | LINC00648,RPS29 | | 0.084 | | intergenic | | 1.40E-09 |
| rs80212697 | 14 | 48744012 | LINC00648,RPS29 | | 0.091 | | intergenic | | 1.40E-09 |
| rs62009439 | 14 | 48744325 | LINC00648,RPS29 | | 0.092 | | intergenic | | 1.40E-09 |
| rs7494430 | 14 | 48745131 | LINC00648,RPS29 | | 0.092 | | intergenic | | 1.40E-09 |
| rs11624500 | 14 | 48745159 | LINC00648,RPS29 | | 0.088 | | intergenic | | 1.40E-09 |
| rs56281472 | 14 | 48745928 | LINC00648,RPS29 | | 0.092 | | intergenic | | 1.40E-09 |
| rs11621878 | 14 | 48746896 | LINC00648,RPS29 | |  | | intergenic | | 1.40E-09 |
| rs12433995 | 14 | 48748897 | LINC00648,RPS29 | | 0.086 | | intergenic | | 1.40E-09 |
| rs60516561 | 14 | 48748923 | LINC00648,RPS29 | | 0.092 | | intergenic | | 1.40E-09 |
| rs62010899 | 14 | 48752657 | LINC00648,RPS29 | | 0.092 | | intergenic | | 1.33E-09 |
| rs118168465 | 14 | 48754404 | LINC00648,RPS29 | | 0.086 | | intergenic | | 1.33E-09 |
| rs11626521 | 14 | 48755795 | LINC00648,RPS29 | | 0.088 | | intergenic | | 1.33E-09 |
| rs75164217 | 14 | 48756060 | LINC00648,RPS29 | | 0.092 | | intergenic | | 1.33E-09 |
| rs78705719 | 14 | 48758585 | LINC00648,RPS29 | | 0.088 | | intergenic | | 1.33E-09 |
| rs62010915 | 14 | 48762169 | LINC00648,RPS29 | | 0.073 | | intergenic | | 1.33E-09 |
| rs62010916 | 14 | 48764717 | LINC00648,RPS29 | | 0.092 | | intergenic | | 1.33E-09 |
| rs62010917 | 14 | 48766698 | LINC00648,RPS29 | | 0.088 | | intergenic | | 1.33E-09 |
| rs62010918 | 14 | 48766964 | LINC00648,RPS29 | | 0.092 | | intergenic | | 1.33E-09 |
| rs12436528 | 14 | 48777981 | LINC00648,RPS29 | | 0.097 | | intergenic | | 1.33E-09 |
| rs17120024 | 14 | 48780899 | LINC00648,RPS29 | | 0.099 | | intergenic | | 1.33E-09 |
| rs62010938 | 14 | 48790841 | LINC00648,RPS29 | | 0.086 | | intergenic | | 1.33E-09 |
| rs28578397 | 14 | 84938133 | NONE,LINC00911 | | 0.094 | | intergenic | | 4.65E-10 |
| rs60078631 | 14 | 84938470 | NONE,LINC00911 | | 0.094 | | intergenic | | 4.65E-10 |
| rs28620444 | 14 | 84942329 | NONE,LINC00911 | | 0.094 | | intergenic | | 4.65E-10 |
| rs116207207 | 14 | 104816344 | KIF26A,C14orf180 | | 0.072 | | intergenic | | 6.03E-21 |
| rs12100834 | 14 | 104817084 | KIF26A,C14orf180 | | 0.1 | | intergenic | | 6.03E-21 |
| rs56808658 | 14 | 104817523 | KIF26A,C14orf180 | | 0.1 | | intergenic | | 6.03E-21 |
| rs77899840 | 14 | 104817736 | KIF26A,C14orf180 | | 0.073 | | intergenic | | 6.03E-21 |
| rs75420965 | 14 | 104818333 | KIF26A,C14orf180 | | 0.11 | | intergenic | | 5.86E-21 |
| rs78290284 | 14 | 104818755 | KIF26A,C14orf180 | | 0.078 | | intergenic | | 5.86E-21 |
| rs74088534 | 14 | 104818864 | KIF26A,C14orf180 | | 0.11 | | intergenic | | 5.86E-21 |
| rs74088536 | 14 | 104818909 | KIF26A,C14orf180 | | 0.11 | | intergenic | | 5.86E-21 |
| rs74088537 | 14 | 104819027 | KIF26A,C14orf180 | | 0.11 | | intergenic | | 5.86E-21 |
| rs74088538 | 14 | 104819095 | KIF26A,C14orf180 | | 0.11 | | intergenic | | 4.87E-21 |
| rs74088540 | 14 | 104819254 | KIF26A,C14orf180 | | 0.11 | | intergenic | | 4.87E-21 |
| rs74088543 | 14 | 104819330 | KIF26A,C14orf180 | | 0.11 | | intergenic | | 4.37E-21 |
| rs74088545 | 14 | 104819616 | KIF26A,C14orf180 | | 0.11 | | intergenic | | 4.49E-21 |
| rs74088546 | 14 | 104819645 | KIF26A,C14orf180 | | 0.073 | | intergenic | | 4.37E-21 |
| rs55678741 | 14 | 104819796 | KIF26A,C14orf180 | | 0.11 | | intergenic | | 4.37E-21 |
| rs56371106 | 14 | 104819867 | KIF26A,C14orf180 | | 0.11 | | intergenic | | 4.37E-21 |
| rs60512647 | 14 | 104820194 | KIF26A,C14orf180 | | 0.11 | | intergenic | | 4.49E-21 |
| rs56199155 | 14 | 104820232 | KIF26A,C14orf180 | | 0.16 | | intergenic | | 5.37E-09 |
| rs74088547 | 14 | 104820616 | KIF26A,C14orf180 | | 0.16 | | intergenic | | 5.33E-09 |
| rs61250295 | 14 | 104822063 | KIF26A,C14orf180 | | 0.11 | | intergenic | | 4.36E-21 |
| rs7144123 | 14 | 104823344 | KIF26A,C14orf180 | |  | | intergenic | | 4.75E-21 |
| rs7143779 | 14 | 104823834 | KIF26A,C14orf180 | | 0.11 | | intergenic | | 4.75E-21 |
| rs8012801 | 14 | 104824914 | KIF26A,C14orf180 | | 0.11 | | intergenic | | 4.75E-21 |
| rs475772 | 15 | 98178270 | LOC101927310,LINC00923 | | 0.082 | | intergenic | | 1.49E-08 |
| rs475797 | 15 | 98178273 | LOC101927310,LINC00923 | | 0.051 | | intergenic | | 7.01E-09 |
| rs288411 | 15 | 98182151 | LOC101927310,LINC00923 | | 0.082 | | intergenic | | 1.49E-08 |
| rs288414 | 15 | 98183830 | LOC101927310,LINC00923 | | 0.082 | | intergenic | | 1.42E-08 |
| rs289503 | 15 | 98186327 | LOC101927310,LINC00923 | | 0.083 | | intergenic | | 1.38E-08 |
| rs289500 | 15 | 98187917 | LOC101927310,LINC00923 | | 0.083 | | intergenic | | 1.38E-08 |
| rs289496 | 15 | 98189497 | LOC101927310,LINC00923 | | 0.084 | | intergenic | | 1.38E-08 |
| rs62023600 | 15 | 99332345 | IGF1R | | 0.0066 | | intronic | | 1.04E-09 |
| rs10438468 | 15 | 100279141 | LYSMD4,DNM1P46 | | 0.088 | | intergenic | | 4.07E-08 |
| rs74805253 | 16 | 2419525 | ABCA17P | | 0.0068 | | ncRNA_intronic | | 9.35E-09 |
| rs117628225 | 16 | 2433747 | ABCA17P | | 0.011 | | ncRNA_intronic | | 3.14E-10 |
| rs79177260 | 16 | 13454596 | SHISA9,ERCC4 | | 0.025 | | intergenic | | 1.03E-08 |
| 16:13474680 | 16 | 13474680 | SHISA9,ERCC4 | | 0.027 | | intergenic | | 1.03E-08 |
| rs12325626 | 16 | 13479614 | SHISA9,ERCC4 | | 0.036 | | intergenic | | 1.03E-08 |
| 16:13481531 | 16 | 13481531 | SHISA9,ERCC4 | | 0.036 | | intergenic | | 1.03E-08 |
| 16:13481627 | 16 | 13481627 | SHISA9,ERCC4 | | 0.036 | | intergenic | | 1.03E-08 |
| rs115286239 | 16 | 13482373 | SHISA9,ERCC4 | | 0.036 | | intergenic | | 1.03E-08 |
| rs114334819 | 16 | 13482375 | SHISA9,ERCC4 | | 0.036 | | intergenic | | 1.03E-08 |
| rs114730118 | 16 | 13484138 | SHISA9,ERCC4 | | 0.036 | | intergenic | | 1.03E-08 |
| rs7206397 | 16 | 13486838 | SHISA9,ERCC4 | | 0.037 | | intergenic | | 1.03E-08 |
| rs7206809 | 16 | 13487028 | SHISA9,ERCC4 | | 0.036 | | intergenic | | 1.03E-08 |
| rs113938109 | 17 | 14938172 | CDRT7,CDRT8 | | 0.011 | | intergenic | | 6.56E-15 |
| rs4439802 | 17 | 47485198 | PHB | | 0.043 | | intronic | | 1.07E-09 |
| rs882030 | 17 | 47485898 | PHB | |  | | intronic | | 1.07E-09 |
| rs882031 | 17 | 47485986 | PHB | | 0.068 | | intronic | | 1.07E-09 |
| rs3986596 | 17 | 47487051 | PHB | | 0.068 | | intronic | | 1.15E-09 |
| rs75408442 | 17 | 47487906 | PHB | | 0.012 | | intronic | | 5.16E-12 |
| rs76056115 | 17 | 47490261 | PHB | | 0.013 | | intronic | | 5.02E-12 |
| rs8073041 | 17 | 47498253 | PHB,LOC101927207 | | 0.073 | | intergenic | | 2.17E-10 |
| rs117899905 | 17 | 47523528 | PHB,LOC101927207 | | 0.0098 | | intergenic | | 2.20E-11 |
| rs17643706 | 17 | 47523871 | PHB,LOC101927207 | | 0.0098 | | intergenic | | 2.20E-11 |
| rs78180261 | 17 | 47524040 | PHB,LOC101927207 | | 0.0098 | | intergenic | | 2.20E-11 |
| rs117035947 | 17 | 47528624 | PHB,LOC101927207 | | 0.0098 | | intergenic | | 2.20E-11 |
| rs79268972 | 17 | 47531241 | PHB,LOC101927207 | | 0.075 | | intergenic | | 8.17E-12 |
| rs77482998 | 17 | 47532356 | PHB,LOC101927207 | | 0.067 | | intergenic | | 7.98E-12 |
| rs75513566 | 17 | 47532434 | PHB,LOC101927207 | | 0.067 | | intergenic | | 1.78E-11 |
| rs16948122 | 17 | 47532513 | PHB,LOC101927207 | | 0.066 | | intergenic | | 1.76E-11 |
| rs62077744 | 17 | 47535773 | LOC101927207 | | 0.12 | | ncRNA_intronic | | 6.39E-12 |
| rs78183039 | 17 | 47536705 | LOC101927207 | | 0.075 | | ncRNA_intronic | | 1.75E-12 |
| rs80194599 | 17 | 47536973 | LOC101927207 | | 0.075 | | ncRNA_intronic | | 1.75E-12 |
| rs2671667 | 17 | 47538935 | LOC101927207 | | 0.13 | | ncRNA_exonic | | 2.18E-12 |
| rs16948132 | 17 | 47539173 | LOC101927207 | | 0.095 | | upstream | | 2.07E-12 |
| rs2412101 | 17 | 47541221 | LOC101927207,NGFR | | 0.074 | | intergenic | | 1.75E-12 |
| rs4789102 | 17 | 72819004 | TMEM104 | |  | | intronic | | 2.78E-08 |
| rs60867224 | 17 | 72959489 | HID1 | | 0.084 | | intronic | | 9.57E-18 |
| rs9907215 | 17 | 72960857 | HID1 | | 0.14 | | intronic | | 1.01E-17 |
| rs9906663 | 17 | 72961013 | HID1 | | 0.11 | | intronic | | 1.03E-17 |
| rs112892463 | 17 | 72966161 | HID1 | | 0.11 | | intronic | | 9.57E-18 |
| rs73365015 | 17 | 72967278 | HID1-AS1 | | 0.11 | | ncRNA_intronic | | 9.57E-18 |
| rs73365017 | 17 | 72967740 | HID1-AS1 | | 0.11 | | ncRNA_intronic | | 9.57E-18 |
| 17:72968974 | 17 | 72968974 | HID1-AS1 | | 0.13 | | ncRNA_intronic | | 9.33E-18 |
| rs57413532 | 17 | 72970451 | HID1-AS1 | | 0.11 | | ncRNA_intronic | | 9.57E-18 |
| rs58976774 | 17 | 72971497 | HID1-AS1 | | 0.11 | | ncRNA_exonic | | 9.57E-18 |
| rs16940792 | 18 | 22110887 | HRH4,LOC729950 | | 0.085 | | intergenic | | 6.65E-22 |
| rs118169451 | 20 | 6034566 | LRRN4 | | 0.0084 | | UTR5 | | 2.72E-24 |
| rs79380602 | 20 | 57111131 | APCDD1L-AS1 | | 0.081 | | ncRNA_intronic | | 3.16E-08 |
| rs79402397 | 20 | 57111257 | APCDD1L-AS1 | | 0.082 | | ncRNA_intronic | | 3.16E-08 |
| rs76959890 | 20 | 57111464 | APCDD1L-AS1 | | 0.084 | | ncRNA_intronic | | 3.16E-08 |
| rs77489329 | 20 | 57111686 | APCDD1L-AS1 | | 0.082 | | ncRNA_intronic | | 3.16E-08 |
| rs7267090 | 20 | 57113140 | APCDD1L-AS1 | | 0.081 | | ncRNA_intronic | | 3.16E-08 |
| rs75161704 | 20 | 57113379 | APCDD1L-AS1 | | 0.081 | | ncRNA_intronic | | 3.16E-08 |
| rs75433891 | 20 | 57113409 | APCDD1L-AS1 | | 0.081 | | ncRNA_intronic | | 3.16E-08 |
| rs10154009 | 20 | 57116050 | APCDD1L-AS1 | | 0.11 | | ncRNA_intronic | | 3.16E-08 |
| rs10154019 | 20 | 57116190 | APCDD1L-AS1 | | 0.1 | | ncRNA_intronic | | 3.16E-08 |
| rs113191532 | 20 | 57117272 | APCDD1L-AS1 | | 0.12 | | ncRNA_intronic | | 3.16E-08 |
| rs57809546 | 21 | 33991774 | C21orf59,SYNJ1 | | 0.013 | | intergenic | | 5.02E-08 |
| rs16993508 | 21 | 37056784 | LOC100506403,MIR802 | | 0.048 | | intergenic | | 9.67E-11 |
| rs73903526 | 21 | 38641161 | DSCR3,DYRK1A | | 0.14 | | intergenic | | 6.88E-10 |
| rs73903529 | 21 | 38644704 | DSCR3,DYRK1A | | 0.14 | | intergenic | | 6.88E-10 |
| rs7278422 | 21 | 38645501 | DSCR3,DYRK1A | | 0.15 | | intergenic | | 6.88E-10 |
| rs6517409 | 21 | 38650500 | DSCR3,DYRK1A | | 0.12 | | intergenic | | 7.03E-10 |
| rs76095513 | 22 | 23769665 | ZDHHC8P1,LOC101929374 | | 0.05 | | intergenic | | 9.49E-10 |
| 22:23770433 | 22 | 23770433 | ZDHHC8P1,LOC101929374 | | 0.05 | | intergenic | | 9.49E-10 |
| rs57021772 | 22 | 23772497 | ZDHHC8P1,LOC101929374 | | 0.055 | | intergenic | | 8.28E-10 |
| rs116834535 | 22 | 23773305 | ZDHHC8P1,LOC101929374 | | 0.049 | | intergenic | | 8.28E-10 |
