# Supplementary figures and images for: Genome-wide association study of prolactin levels in blood plasma and cerebrospinal fluid
Source: BMC Genomics. 2016 Jun 29;17(Suppl 3):436. doi: 10.1186/s12864-016-2785-0 (PMC4943503; doi:10.1186/s12864-016-2785-0)

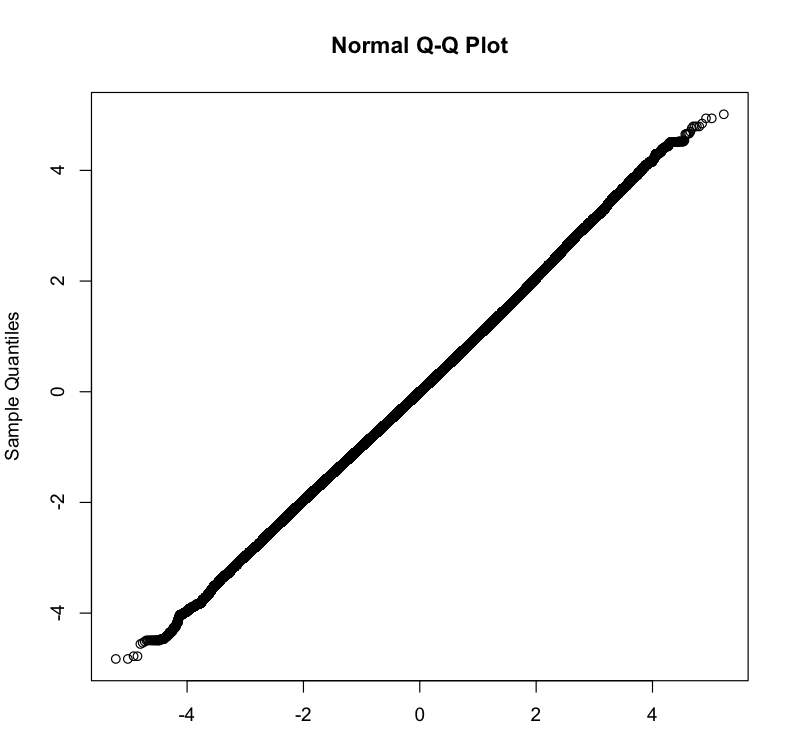

Supplement: Additional file 3: — File contains a Q-Q plot of the plasma data used in this study. (DOCX 74 kb) [file 12864_2016_2785_MOESM3_ESM.docx]

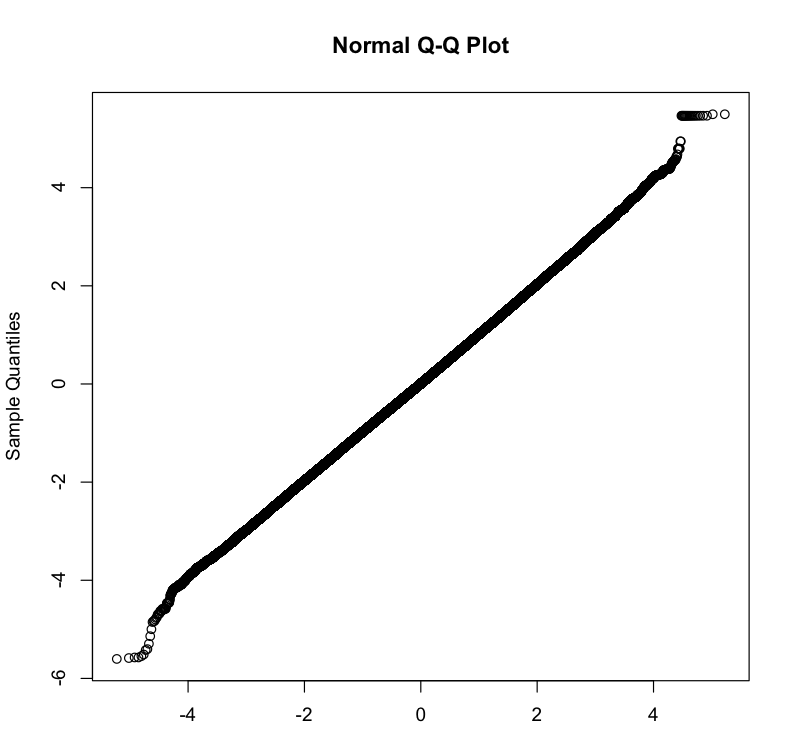

Supplement: Additional file 4: — File contains a Q-Q plot of the CSF data used in this study. (DOCX 76 kb) [file 12864_2016_2785_MOESM4_ESM.docx]
